# Regional impacts on decarbonisation under evolving financing conditions for energy technologies

**DOI:** 10.1038/s41467-026-73522-1

**Published:** 2026-05-19

**Authors:** Natasha Frilingou, Dirk-Jan Van de Ven, Jon Sampedro, Russell Horowitz, Clàudia Rodés-Bachs, Thomas Nikolakakis, Anastasios Karamaneas, Kimon Georgiou, Konstantinos Koasidis, Shivika Mittal, Charalampos Platias, Conall Heussaff, Christoph Bertram, Alexandros Nikas

**Affiliations:** 1 Energy Policy Unit, School of Electrical & Computer Engineering, National Technical, University of Athens, Athens, Greece; 2 Basque Centre for Climate Change (BC3), Leioa, Spain; 3 IKERBASQUE, Basque Foundation for Science, Plaza Euskadi 5, Bilbao, Spain; 4 CICERO Center for International Climate Research, Oslo, Norway; 5 Department of International and European Studies, Panteion, University of Social & Political Sciences, Athens, Greece; 6 Bruegel, Brussels, Belgium; 7 Center for Global Sustainability (CGS), School of Public Policy, University of Maryland, College Park, MD USA; 8 Potsdam Institute for Climate Impact Research (PIK), Member of the Leibniz Association, Potsdam, Germany

**Keywords:** Climate-change mitigation, Energy policy, Climate-change mitigation, Social sciences

## Abstract

Energy-sector decarbonisation requires large-scale investment in low-carbon technologies, yet only a limited share flows to low- and middle-income countries, partly due to higher financing costs and perceived risks. Most modelling exercises do not fully account for how the cost of capital may vary across regions and technologies, potentially influencing policy insights. We examine how plausible, expert-informed long-term trends in de-risking clean energy and increasing risks for fossil fuels could shape decarbonisation pathways, using an empirical dataset differentiated by country and technology. We also evaluate a “corrective justice” policy that taxes corporate windfall profits and redistributes revenues to support low-carbon investments in higher-risk regions. Results suggest that incorporating differentiated cost-of-capital trajectories may improve mitigation outcomes and help narrow the gap between current commitments and long-term climate targets, while indicating potential underestimation of risks associated with bioenergy-based negative emissions technologies in mitigation scenarios for high-income nations.

## Abstract (web summary)

Differentiated financing costs impact decarbonisation pathways: de-risking clean energy and/or increasing fossil-fuel risks can reduce emissions; widening regional finance gaps may slow transitions. Recycling windfall profits could support low- and middle-income countries.

## Introduction

Decarbonising the economy should encompass global action to address fundamental economic disparities and overcome the climate investment trap faced by low-income nations^1^. It is estimated that $2.8 trillion was invested in energy in 2023, with over $1.7 trillion going to clean energy, while the remainder towards fossil fuels instead^2^. Modelled pathways achieving the Paris Agreement global temperature goal of limiting warming to 1.5 °C above pre-industrial level require cumulative investment across the entire global energy system of over USD 6.7 trillion annually by 2030^3^. The climate financing gap also encompasses a persistent geographic misallocation of global capital: nearly all recent growth of investments in clean energy is directed towards advanced economies and China. Contrarily, capital flows have remained flat in middle- and low-income economies, accounting for only 15% of clean energy investments, despite these countries representing two-thirds of the world population and contributing to one-third of global GDP^4^. Although climate finance flows overall have been sparing in regions with lower access to climate finance^5^, post-COVID debt, high inflation and interest rates, and the rise of protectionist industrial policies in major economies—such as the US Inflation Reduction Act (IRA)—have redirected much of the available clean-energy investment toward domestic markets, indirectly constraining capital flows to low and middle income countries (LMICs)^6^. Low-carbon technologies require large upfront investments, unlike fossil-fuel technologies, where fuel costs make up the dominant part of the lifecycle costs^7^. The private cost of capital—i.e., the discount rate employed when evaluating private investments—becomes essential to assessing investment choices among various alternatives, such as different power generation technologies^8^. The cost of capital is thus used in investment appraisals, such as in levelised cost of energy (LCOE) calculations, in which the capital-intensive nature of low-carbon technologies implies high sensitivity to cost of capital rates^9^. It is thus a major determinant of the cost of deploying renewable power generation technologies, as an increase in the cost of capital raises financing expenses, thereby posing challenges in achieving attractive risk-adjusted returns. In advanced economies, capital expenditures (e.g., land and equipment acquisition) typically constitute the most important chunk of total clean energy project expenses, while in LMICs economies it is financing costs, i.e., the cost of capital of those projects, that take the lead^4^. Although clean energy technology costs have been decreasing in the last decades, in most LMICs economies political, regulatory, and macroeconomic risks lead to difficulties in securing the required finance^10,11^. Reducing the costs of capital in LMICs economies would thus benefit investors and boost national mitigation efforts, while enhancing the overall affordability of energy transitions.

Reliable data and an improved understanding of the composition and long-term evolution of the cost of capital and its drivers are essential to develop support mechanisms and market designs that appropriately consider varying technology and country risks^12^. These are commonly represented in the weighted average cost of capital (WACC) that considers the proportion of debt and equity financing in a company’s capital structure and is used as a hurdle rate to assess the desirability of investment projects^13^. In essence, WACC is a weighted average of the cost of debt and the cost of equity, where the weights are determined by the share of each in a company’s total capital^14^. In case of energy projects characterised by elevated perceived risk, such as those in politically unstable regions or involving newer and highly uncertain technologies, WACC values are higher, signifying that investors require greater returns to offset the higher risk of potential financial losses^15^. Rising interest rates could thus greatly undermine the economic feasibility of investments in renewable energy sources (RES)^16^.

There have been recent endeavours to create datasets of empirically grounded WACC values for power-sector technologies: the International Renewable Energy Agency (IRENA) has developed such a dataset at the global level^12^ including green hydrogen^17^, the International Energy Agency (IEA) tracks the cost of capital for clean energy projects in LMICs economies^18^, while research efforts have focused on putting together regionally disaggregated WACC values for a subset of electricity production technologies in the EU^19,20^, Africa^21,22^, and globally^11,23^. These datasets, however, fall short on delivering a set of plausible long-term projections on the evolution of WACC values. As such, most model-driven scenario exercises (i.e., using integrated assessment models; IAMs), among those used to underpin climate change mitigation strategies, often use uniform WACC values across both technologies and regions over time, which can disproportionately impact mitigation pathways for low-income economies^11^ and may bias policy insights^24^.

In a recent study, Calcaterra et al.^23^ contributed to bridging part of this gap, by developing an empirically calibrated WACC dataset by region and technology, which was then parsed in the underlying assumptions of IAMs and considering financial learning as well as the possibility of gradual cost-of-capital convergence across the globe, to cover ground in the WACC long-term evolution space. Drawing on IRENA’s approach towards building short-term projections for the costs of capital^12^ that are partially informed by expert elicitation, here we combine the empirical estimates of baseline WACC values in Calcaterra et al.^23^ with an expert-based approach—a process found to yield narrow longer-term uncertainty^25^—to produce plausible, forward-looking, long-term projections of evolving costs of capital over time. Our study further builds on and expands the baseline dataset^23^ with data on hydrogen^17^ and, using a global IAM, shifts the focus to regional decarbonisation pathways under alternative technological and policy choices, with particular emphasis on derisking clean energy and risking fossil-fuel technologies—exploring how divergent WACC futures could emerge from policy choices, similar to the logic of Battiston et al.^26^.

Critically, our analysis also considers today’s economic context in two ways. First, we conduct a sensitivity analysis to additionally account for inflation in our scenario exercise. Second, acknowledging that the recent surge in energy prices has increased firms’ input costs and households’ energy expenditure, leading to substantial windfall profits^27^, taxing which has been increasingly proposed to fund the financing gap by underwriting capital market for low-carbon investments in LMICs economies^28,29^, our study incorporates a ‘corrective justice’ dimension^30^: we consider an international policy instrument of risk underwriting for low-carbon investments that taxes those windfall profits and then assess the impact of distributing this revenue as subsidies towards high-WACC regions.

## Results

### Co-designed long-term projections of costs of capital

To explore how different developments of WACC values across technologies and countries may impact decarbonisation, the empirically grounded WACC values from Calcaterra et al.^23^ and IRENA^17^ constitute our starting point. Acknowledging that the evolution is largely uncertain and depends among others on risks associated with country contexts, currencies, technology and financial maturity, political developments, etc., projecting these values forward was the core objective of an online workshop, held in October 2023, between the research team and 20 experts. The pool of experts included policymakers, financial sector executives, and representatives of non-governmental organisations, who—following an in-depth discussion on uncertainty factors and how these may play out—were also asked to fill out a survey on how perceived costs may evolve for different technologies and countries around the world.

We then proceed to design alternative scenarios of such a future. The first set of scenarios is based on the results of the survey, featuring the precise responses of our experts (scenario *Sv* in the figures below). This is further broken down into two additional variants to disaggregate the impact of two tendencies observed in the survey responses, namely increased risk of fossil fuel investments and derisking of renewable technologies. As such, one variant sticks to the experts’ opinions on fossil fuels becoming less competitive in the future; to reflect this, we use the survey values for these technologies, while keeping non-fossil technologies constant over time in line with the employed empirical dataset (scenario *Sv-F*), overall reflecting a world of fossil fuel divestment. Second, we make the opposite projection, using the WACC value trajectories provided by the experts for the non-fossil fuel technologies to portray a world of derisking non-fossil fuel technologies: WACC values for renewables, green hydrogen, hydropower, and CCS decrease, values for nuclear and biomass increase, while fossil-fuel values are kept constant and identical to the empirical dataset (scenario *Sv-NF*). In a fourth scenario, we reflect a fragmenting world, in that a variety of economic, financial, and geopolitical factors lead to increasing divergence—essentially supplementing the convergence scenario of Calcaterra et al.^23^—of costs of capital between high-income countries and low- and middle-income countries (LMICs) (scenario *Frag*). A final scenario is designed to represent the introduction of a policy measure that distributes the revenues of a tax on windfall profits for the year 2022 across the globe and compensates RES deployment, in countries with the highest needs and perceived risks, and on top of currently implemented policies (scenario *WF*).

Three baseline trajectories are also defined, against which scenarios are benchmarked to allow quantifying the impact. The Current Policies (CP) baseline (scenario *B-CP*) represents the level of short-term ambition that is likely to be materialised through actual policies or concrete policy targets of all regions until 2030^31^; post-2030, current policies are assumed to remain in place as “constant” or “minimum” bounds on effort. The Nationally Determined Contributions (NDC) baseline (scenario *B-NDC*) also represents current policies explicitly, on top of which NDCs are enforced until 2030; the rate of change in emissions intensity of GDP in each region from 2020 to 2030 is kept constant onwards—a method proposed by Fawcett et al.^32^ and Vandyck et al.^33^ to assess the long-term implications of NDCs. To increase the realism of how emissions reductions take place in all our scenarios, current policies are represented explicitly in both the *_CP* and *_NDC* baselines. NDC targets are based on a direct interpretation of countries’ unconditional Paris Agreement pledges—or the less ambitious range for pledges where NDC targets are given in ranges—as announced by 2023. Moreover, all baseline trajectories assume the non-uniform risk perceptions of the employed empirical WACC dataset differentiated by region and technology^17,23^. Finally, our expert-driven pathways are compared against NDC targets by 2030 and longer-term emission targets (LTTs) (scenario *B-LTT*) to examine how potential financial sector dynamics can decrease CO_2_ emissions towards delivering on long-term ambition.

Our scenarios are designed to replicate real-world policies and regional emission patterns up to 2020 and start diverging from 2025 when WACC variations are introduced. Model assumptions have been updated to reflect the latest trends, including population drawn from the EUROPOP2019^34^ database and the short-term economic outlook of the International Monetary Fund’s World Energy Outlook (IMF WEO) of October 2023 until 2027^35^, extrapolated to 2050 according to the SSP2 socioeconomic pathway reflecting historic trends.

A high-level summary of all scenarios is available in Table 1; more information can be found in 'Methods'.

**Table 1:** Scenario protocol

Name	WACC trends and climate policies assumptions
Β-CP	Based on the current portfolio of announced emissions reduction policies and credible policy targets until 2030 in G20 countries. Post-2030 action is modelled by measuring the average rate of change in emissions intensity of GDP from 2020 to 2030 in each region and assuming emissions-intensity reduction rates remain the same after 2030. Applied policy targets until 2030 are maintained as minimum levels beyond 2030 to avoid backtracking of achieved policies. Empirical WACC values are used and kept fixed for the entire time horizon.
B-NDC	Based on 2030 emission targets pledged in NDCs submitted or announced by June 2023, capturing all mitigation ambition updates during and after COP26 in Glasgow, implemented on top of current policies. In model regions with current policies exceeding NDC mitigation targets, no additional emission constraints are applied; emissions reductions are therefore never less ambitious than implied in current policies. Empirical WACC values are used and kept fixed for the entire time horizon.
B-LTT	For regions that have expressed an LTT—e.g., net-zero commitments or other targets for 2050 or later—emission constraints linearly decline from 2030 levels from the NDC target towards the LTT. For regions without LTTs, post-2030 the applied policy targets are maintained as minimum levels beyond 2030 to avoid backtracking of achieved policies. Empirical WACC values are fixed for the entire time horizon.
Sv	Empirical WACC values are used as baseline (2020), while future WACC values change for all technologies linearly in 2018–2050, according to our survey results (Table S1). Carbon prices are taken from *B-NDC*.
Sv- inflation	The same as *Sv*, but with an updated risk-free rate according to the latest available estimates at the time of our analysis (4.018% as of October 2024).
Sv-F	Empirical WACC values are used as baseline (2020), while future WACC values for fossil fuels increase linearly in 2018–2050, according to our survey results (Table S1). Carbon prices are taken from *B-NDC*.
Sv-NF	Empirical WACC values are used as baseline (2020), while future WACC values for RES, green H_2_, hydro, and CCS decrease, and those for biomass and nuclear increase linearly in 2018–2050, according to survey results (Table S1). Carbon prices are taken from *B-NDC*.
Frag	Empirical WACC values are used as baseline (2020), while future WACC values of high-income countries further diverge from those in LMICs, linearly in 2018–2050: for high-income countries (incl. China), WACC values converge to the 25th percentile of the average baseline empirical WACC value per technology; for LMICs, WACC values converge to the 75th percentile of the average baseline empirical WACC value per technology (Table S1). Carbon prices are taken from *B-NDC*.
WF	Empirical WACC values are used as baseline (2020) and kept fixed for the entire time horizon. The subsidy allocation by country is calculated as a linear combination of three shares: (a) total subsidy to GDP per capita, (b) proportional share of each country’s GDP | PPP to total GDP | PPP, and (c) proportional share of WACC by country and technology; low-risk regions (i.e., average WACC < 6%) are excluded (Supplementary Data S2–S4; Table S3; 'Methods'). The budget in each region is used to construct new RES capacity at the same costs of capital as in the West (see Table S3). Carbon prices are taken from *B-CP*.

**Fig. 1:**
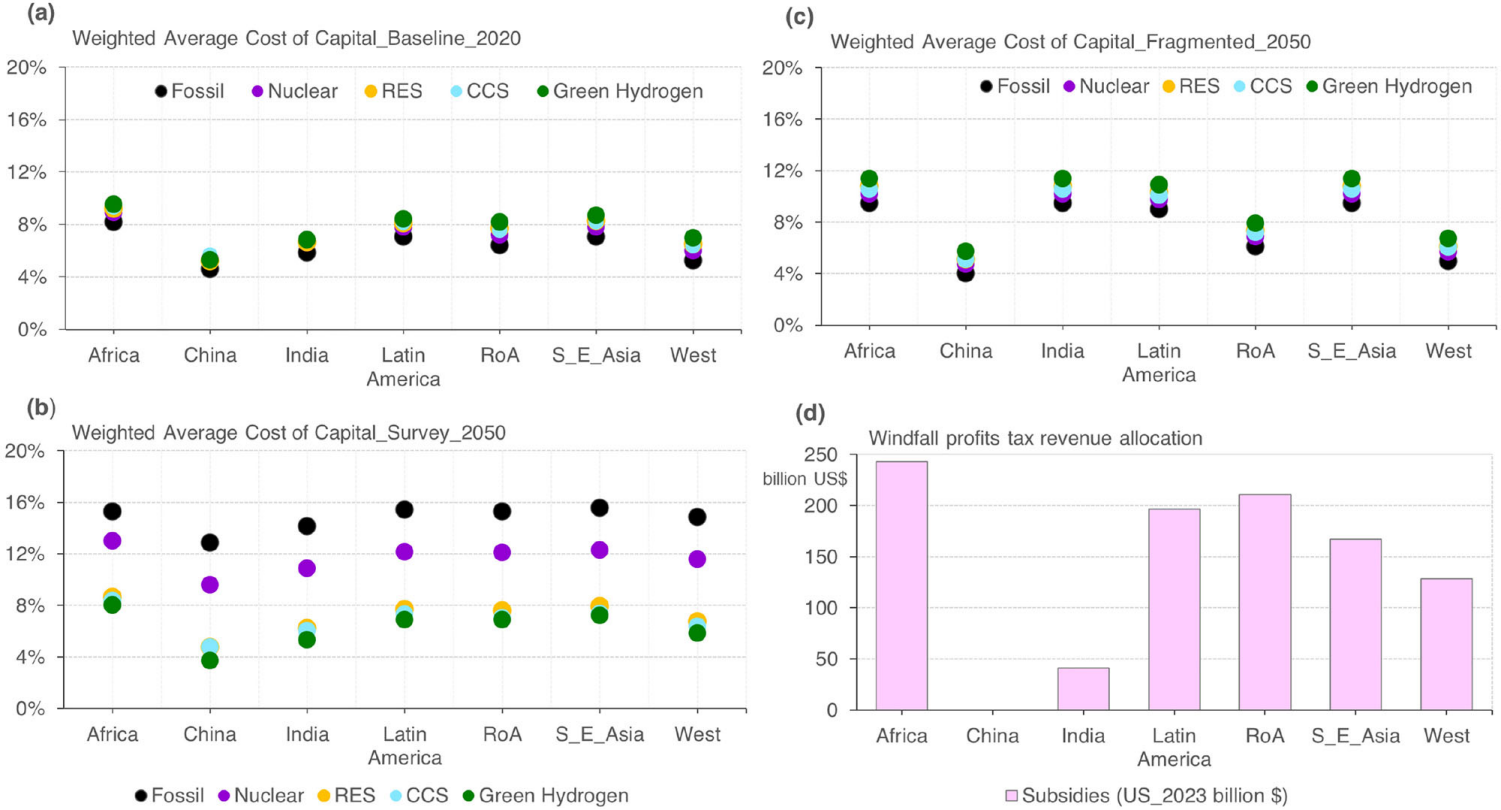
Baseline and projections of Weighted Average Cost of Capital (WACC) values per scenario and windfall profits. WACC values aggregated by aggregated region and technology (fossil fuels, renewables, nuclear, CCS, and green hydrogen), **a** in the baseline year; **b** in 2050 based on the survey results; **c** and in 2050 for the Frag scenario; RES Renewable Energy Sources, CCS Carbon Capture and Storage. **d** Windall profits tax revenues allocation by region in US_2023_ billion $. Scenarios are explained in Table 1, while aggregated regions in Table S2.

Figure 1 illustrates baseline and future (aggregated) WACC values by region and technology per scenario, along with the distribution of windfall profits allocation by region. Variation in empirical region-specific WACC values (Fig. 1a) is mainly attributed to country-related risks, underdeveloped local financial systems, and macroeconomic stability^23^. The *Frag* scenario (Fig. 1b) represents a clear divide among low-middle- and high-income regions compared to the baseline, while in the survey (*Sv*) scenario (Fig. 1c), stakeholders indicated a substantial increase in future WACC values for both fossil fuels and nuclear power, and a moderate decrease for renewables, CCS, and green hydrogen. For the implementation of the windfall profit taxation scenarios (Fig. 1d), we assumed a total revenue of USD_2023_ 990.00 billion globally^29^.

### Changes in the energy system

By building on and expanding empirical values reflecting investment risks, our modelling exercise suggests, especially for low-income economies (e.g., in Africa), that policies aimed at derisking renewables could prove an effective lever for reaching net-zero targets. However, regions with extensive fossil-based infrastructure would face structural challenges, with declining costs of capital driving clean energy investments but not sufficiently to entirely replace fossil fuels (e.g., in China). Temporal changes highlight limited additional reductions by 2030 across all regions compared to *B-NDC*, which is indicative of the relatively small changes in WACC values compared to the baseline (Fig. 1b, c).

**Fig. 2:**
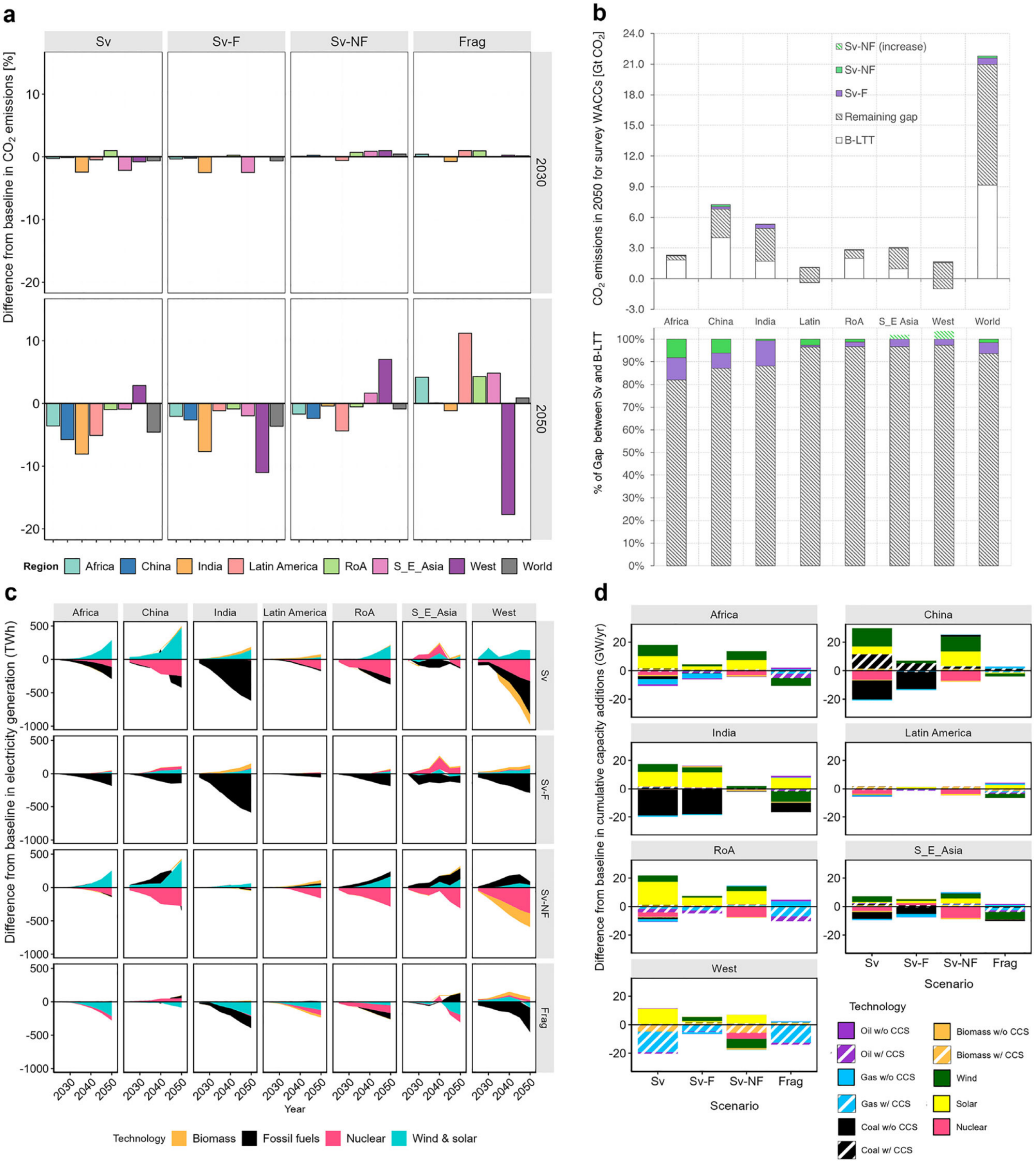
Regional energy-system implications of different Weighted Average Cost of Capital (WACC) projections on top of Nationally Determined Contributions (NDCs). **a** Percentage change of CO_2_ emissions from energy and industrial processes by scenario and region compared to *B-NDC* in 2030 and 2050. **b** Gap between *B-NDC* and *B-LTT* CO_2_ emissions in 2050 when survey WACCs are applied, Fossil & Non-Fossil contributions are shown separately. **c** Electricity generation by technology relative to *B-NDC*. **d** Cumulative capacity additions by scenario, technology, and region during 2025–2050 relative to *B-NDC*; CCS: Carbon Capture and Storage. Scenarios are explained in Table 1, while aggregated regions in Table S2.

The highest emissions decrease (Fig. 2a) would happen in the case of fossil fuel divestment (*Sv-F*) (i.e., reflected through higher fossil-fuel WACC values. Fossil (coal, oil & gas) secondary electricity would be replaced by renewables, biomass, and nuclear—with the latter more evident in China (+ 65TWh RES and +46TWh nuclear in 2050 relative to *B-NDC*) and countries in South and East Asia ( + 95 TWh nuclear and +22TWh biomass in 2050 relative to *B-NDC*) (Fig. 2c). This is also the scenario with consistent decarbonisation gains across the globe in the longer-term, although Western countries (−11%) and India (−8%) showcase the largest achievements in terms of mitigation, with extensive reductions in fossil electricity and capacity additions mostly in solar and wind. Countries in Africa and South and East Asia would exhibit smaller emission reductions (−2%), indicative of slower energy transitions rather than transformative shifts due to economic or infrastructure constraints.

Derisking renewable energy investments (*Sv-NF*)—e.g., by reallocating, sharing, and/or reducing existing potential risks associated with those investments^36^—would cut global CO_2_ emissions through capacity additions in RES (Fig. 2d) and increased green electrification. Results highlight the impact of rising WACC values on the deployment of nuclear power generation; its high upfront costs and long lead times^37^ would contribute to the observed important drop in its share in the energy mix in regions with high baseline nuclear capacity. Despite the lower risk of renewables, emissions would increase in the West for a multitude of reasons. Notably, fossil fuels would remain cost-competitive and still get deployed, the use of nuclear energy and BECCS (Bioenergy Carbon Capture and Storage) would decrease, and a shift from wind to solar would be observed, with the latter two reasons contributing to a decrease in total electricity produced, hampering electrification efforts in other sectors (Fig. 2c, d).

Future WACC pathways according to our survey results (*Sv*), which essentially combine the assumptions of fossil and non-fossil pathways discussed above, could contribute toward bridging the 2050 emissions gap between extrapolated NDCs and long-term targets (*B-LTT*) by as much as 18% in Africa, and 6% globally (Fig. 2b); interestingly, the largest chunk of this reduction (5%) could be attributed to increased fossil-fuel risks alone. A wide gap would however remain, indicating that lowering WACC values alone is not sufficient to scale up decarbonisation in line with long-term emission targets. The opposite is observed in the West, as emissions increase most notably in the US owing to lower deployment of negative CCS-based technologies (i.e., BECCS with the risk of biomass almost doubling in the country, and DACCS—see Fig. S1). In regions like Latin America, where the RES share in electricity generation is already high^38^ and grows further in the baseline scenario, lower WACC values in these technologies would only moderately increase solar and wind electricity, eventually contributing to bridging the emissions gap towards their LTTs by about 3%.

Particularly for this scenario, we also perform sensitivity analysis, acknowledging that, in today’s inflationary environment, the estimation of the risk-free rate—which is defined as the nominal yield on a 10-year US treasury bond^23^ and serves as a benchmark for a wide range of interest rates, including those for mortgages, corporate bonds, and other loans—would be affected. We thus update the value of the risk-free rate to the latest available estimates (4.018%, as of October 2024) and revisit our modelling exercise for the survey (*Sv*) scenario, in the form of sensitivity analysis, to explore the impact of an increased global risk-free rate on modelling outputs (Fig. S2). By definition, an increased global risk-free rate would raise the WACC values for all technologies (Table S1), which would in turn hinder their competitiveness. More capital-intensive technologies (notably nuclear power) would markedly decline until 2040, but subsequent growth of renewables would compensate for the large decrease of nuclear power and manage to even out CO_2_ emissions by 2050.

Finally, a world where investment risks between high- and low-/mid-income countries further diverge (*Frag*) would instead showcase decreased deployment of renewable energy for power generation in most low-income nations in Africa and Asia, with coal and/or gas gaining ground and leading to a 5–10% increase in CO_2_ emissions (Fig. 2, scenario *Frag*). Despite delivering the highest emission reductions in the West, which may be attributed mainly to the non-increasing risk of nuclear power and thus its continued use in Europe and the USA, this scenario would be the most carbon-intensive for the rest of the world, overall leading to a (marginal) increase in global CO_2_ emissions. Decisions for production, consumption, and investment in our model are only based on the prices of each period, meaning that decarbonisation is proportional to investment, and thus scenarios with higher WACC values for green technologies tend to showcase relatively limited mitigation^39^.

### Regionally differentiated impacts on electricity investments and prices

It is estimated that annual capital investment in clean energy in LMICs needs to increase from $270 billion today to $870 billion by the early 2030 s to align with national climate and energy commitments^4^. Our assumptions on WACC evolution in 2030 are not markedly different from the baseline for clean energy technologies, averaging a decrease of 6% in solar and rooftop PV, 3% in off/onshore wind, and 4% in green hydrogen for LMICs. Hence, without additional enabling measures, WACC values alone cannot drive the necessary threefold increase in investments by as early as 2030.

**Fig. 3:**
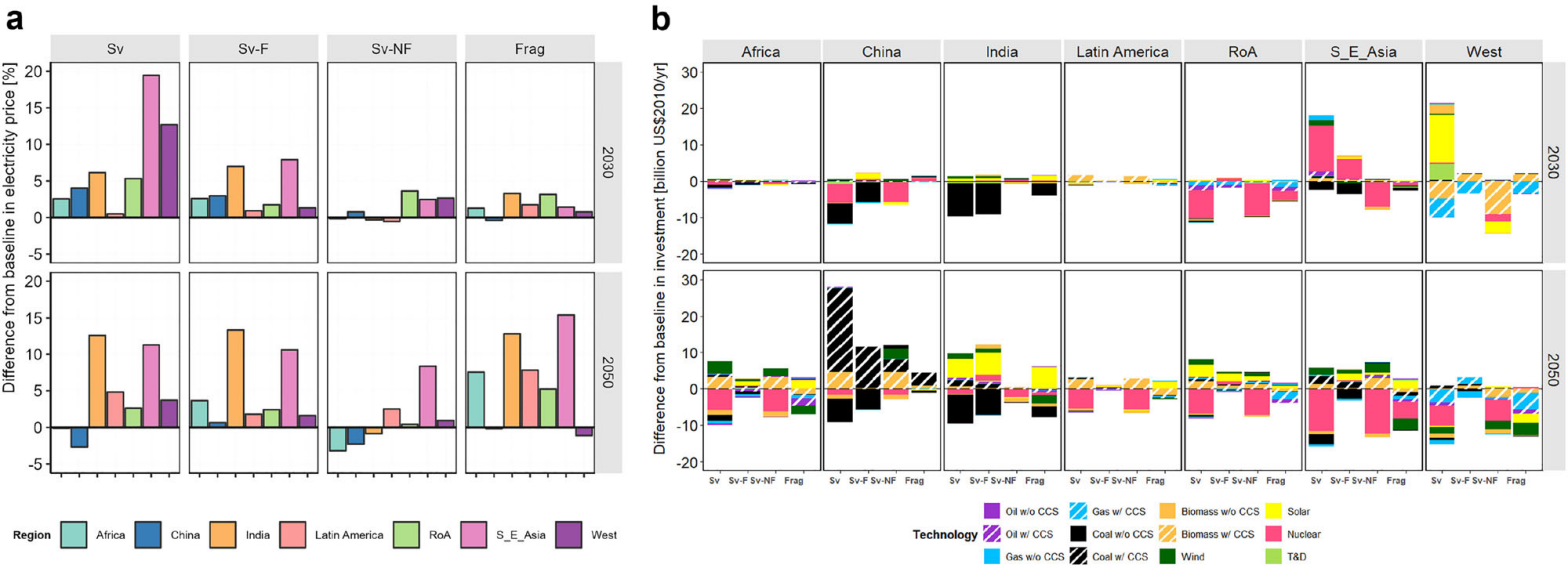
Regional implications of different Weighted Average Cost of Capital (WACC) projections for electricity prices and energy investments, on top of Nationally Determined Contributions (NDCs). **a** Percentage change of weighted average electricity prices by final electricity consumption per region and scenario (Supplementary Data S6) compared to *B-NDC* in 2030 and 2050. **b** Difference of investments in electricity supply technologies compared to *B-NDC* by scenario and region, in 2030 and 2050. T&D Transmission & Distribution, CCS Carbon Capture and Storage. Scenarios are explained in Table 1, while aggregated regions in Table S2.

A higher cost of capital of fossil fuels technologies would partially discourage investment in new coal, gas, and oil capacity due to lower expected returns. Despite the increasing WACC values, fossil fuel technologies would still be deployed, leading to electricity price spikes across all regions. The increased LCOE of fossil fuels would feed directly into electricity market prices—especially in fossil-dominated grids such as India’s, where prices could rise by up to 15% compared to the baseline (Fig. 3a, *Scenario Sv-F*). This would in turn reduce demand and thus investments towards fossil-fuel electricity infrastructure (Fig. 3b).

Lower WACC values could boost investments in capital-intensive renewables, especially in China (+$6.4 billion), other Asian countries (South and East Asia +$5.1 billion, +$3.1 billion elsewhere), and Africa (+$3.3 billion), in 2050 (Fig. 3b, *Scenario Sv-NF*). In regions with an already favourable investment framework (e.g., in the West), where RES investments are less risky, an incremental risk reduction of WACC values would not incur important investment shifts. However, in combination with riskier fossil fuels (*Sv*), more than $13 billion/yr investments could flow towards solar, and $4.6 billion towards transmission & distribution in 2030—a much quicker response to costs of capital compared to other regions where investments occur mostly towards mid-century. Renewables’ falling WACC values would moderately reduce electricity prices towards 2050 in China, India, and Africa, due to the simultaneous increase of WACC values for nuclear—a capital-intensive technology that is highly susceptible to such variations (Fig. 3a, *Scenario Sv-NF*). Financing risks would affect the affordability of nuclear power investments all over the world, more prominently in South and East Asia, where investments could drop by $12 billion in 2050.

We also observe an increase in investments related to CCS, with coal in Asia (amounting to +$12.5 billion in total)—including the two highest emitters from coal in power generation (China and India)—and with gas in the West (+$1.9 billion), despite the rising cost of capital of fossil fuel technologies; as new fossil-fuel capacity additions become riskier while CCS becomes more competitive, investments are increasingly directed towards new coal capacity with CCS (Fig. 2d). Similarly, in case of derisking non-fossil fuel technologies, our analysis suggests a large flow of investments towards BECCS ($14.6 billion total) in most parts of Asia, Africa, and Latin America (Figs. 3b and S3), even if biomass WACC values instead increase over time.

Deepening inequality, in cost-of-capital terms (*Scenario Frag*), would yield the highest electricity price increases in all regions, except for Western countries (where WACC values do not increase) and China, hampering electrification of end uses as well as overall investments in the electricity sector (Fig. 3a, b, *Frag*), and leading to higher emissions in 2050, especially in regions with high price spikes (Africa, Latin America, and all Asian countries apart from China).

### Making use of taxes on windfall profits to reduce investment risks

Windfall profits are gains that arise not from a company’s deliberate strategy or planned actions, but from unexpected shifts in market conditions that could not have been anticipated when the original investment was made. Such windfall profits may constitute the full economic rent (i.e., excess profit) or only a portion of it, with the remainder reflecting firm- or location-specific advantages. Economists argue that taxing economic rents is efficient because it targets only those returns above what is necessary to justify the investment, leaving the baseline incentives intact^40^. Governments often levy windfall profit taxes to meet urgent budgetary needs—such as funding relief measures to shield consumers from soaring living costs amid high inflation—or to capture what are seen as unjust gains by industries during unexpected events, reallocating those unexpected earnings for the public benefit^41^. Obligations for reinvesting windfall profits of fossil fuel energy revenues in energy transition technologies are among the fiscal measures proposed to deal with the energy crisis and accelerate the energy transition^3^. The collected tax revenue could be distributed to vulnerable households or hard-hit firms, with the aim to either reduce prices or support income. In this study, we assume that the revenue of windfall profit taxes is distributed as subsidies to clean energy projects towards high-WACC regions, with the aim of lowering perceived investment risks.

Policies towards cost-of-capital convergence among high-income countries and LMICs could yield positive outcomes in terms of energy accessibility, affordability, and sustainability, as well as both intra- and inter-generational equity^23^. Moreover, distributive justice considerations in global climate mitigation could require annual interregional flows of at least PPP (2015) $570 billion in the near-term, only for mitigation efforts^42^. Here, we aim to incorporate such considerations in our mitigation scenarios in the form of compensatory payments^30^ towards countries facing high RES (solar, wind, green hydrogen) costs of capital. Subsidies are allocated as a function of three indicators (see Methods): (a) countries’ living standards measured as the share of gross domestic product (GDP) per capita for the year 2018 (USD_PPP, 2017_ per capita) to the total available subsidy, so that countries with lower GDP per capita can get higher subsidies; (b) countries’ WACC values for clean energy technologies (solar, wind, green hydrogen), so that countries with very low-WACC values—i.e., <6% (IRENA, 2023)—do not receive subsidies; and (c) countries’ capability, measured as a share of countries’ GDP_PPP_ to global GDP_PPP_. Note that subsidies by region (Fig. 1d) are distributed equally between 2025 and 2030 and modelled on top of current policies (scenario *WF*), instead of NDCs, as we are primarily interested in boosting near-term mitigation investments for which the existing policy framework is more important.

**Fig. 4:**
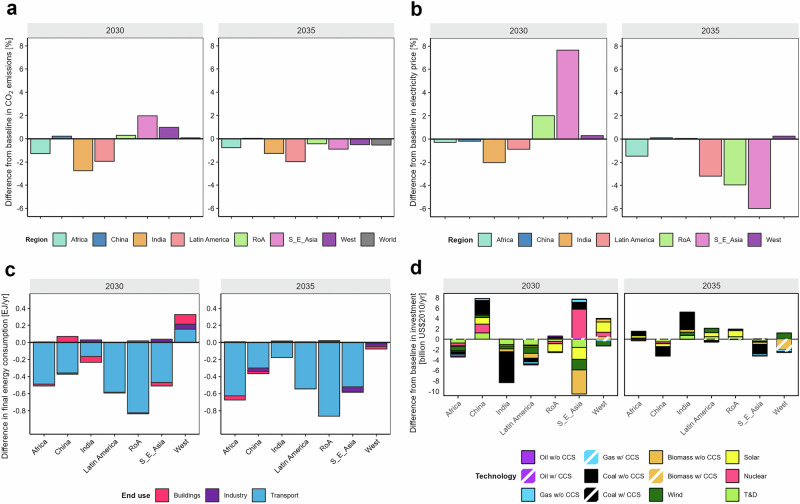
Regional implications of a corrective justice policy that distributes revenues from a global windfall profit tax, on top of current climate policies. **a** % change of CO_2_ emissions from energy & industrial processes compared to *B_CP* in 2030 & 2035; **b** % change of electricity prices compared to *B_CP* in 2030 & 2035; **c** absolute difference of energy consumption for end-use sectors in 2030 & 2035; **d** absolute difference of investments in power sector technologies in 2030 and 2035; CCS: Carbon Capture and Storage. Scenarios are explained in Table 1, while aggregated regions in Table S2.

Our results indicate that subsidies alone could not drive clean energy investments when fossil fuels remain competitive; they would, however, lower both CO_2_ emissions and electricity prices in the mid-term in regions receiving high amounts (Fig. 4a, b; Africa, Latin America, and Asia—except for China and India). As subsidies make electricity cheaper, we observe a higher electrification of transport, which drives demand reduction in Fig. 4c. Other end-use sectors are not greatly impacted, while in regions with high electricity use in buildings and industry (China, West) there is in fact higher final energy demand when electricity is subsidised. In terms of investments, without considering future technological risks, subsidies would also be directed towards coal (in China and RoA) as well as nuclear (in China and South and East Asia), thereby reinforcing today’s electricity regime (Fig. 4d).

## Discussion

Despite their relatively higher vulnerability to climate impacts, LMICs lack the financial resources to act on climate. Currently, global climate finance flows are greatly insufficient compared to regional and sectoral needs and must increase by a factor of 3–6 to meet average annual needs by 2030^43^. Promised financial support from high-income countries has been delayed and inadequate, and the climate finance deal struck at COP29 did not meet the expectations of those in need^44^. Ramping up climate investments in LMICs represents an important opportunity for wealthy economies too^45^ and, unless financial flows scale up urgently to support the necessary investment push, the world’s climate objectives are at risk^46^.

Low-carbon energy transitions entail an important reallocation of energy spending, from dollar-denominated, globally traded commodities like oil and gas to capital-intensive investments in renewable technologies. Despite relatively stable lifetime costs, the latter introduce new risks associated with pricing in local currencies, dependence on domestic demand and counterparties, and limited maturity of risk management mechanisms—particularly in LMICs^47^. Although political decision-making and public financing can have a strong impact on incentivising private-sector climate capital^48^ and pushing expected returns down^49^, here we have taken aim directly at market mechanisms, as reflected in the cost of capital—a critical benchmark for investors upon evaluating risk and return preferences as well as an effective lever for financial flows to influence prices and choices in the real energy economy^50^. We particularly focused on the weighted average cost of capital, which constitutes a large part of levelised energy costs^51^ and can impact the affordability of energy transitions, especially in LMICs.

Calcaterra et al.^23^ recently highlighted that representing real-world costs of capital across low-carbon technologies and regions is critical to more accurately assess the financing cost of decarbonisation around the world that is implied in mitigation pathways compatible with climate policies and pledges. Our study, in turn, advances the literature in four ways: first, it develops forward-looking, expert-elicited trajectories of the cost of capital rather than using static baseline values; second, in doing so, it expands technology coverage to include hydrogen values and reinforce the added value of the original dataset; third, it embeds financial policy instruments such as risk-underwriting subsidies funded through windfall-profit taxation to explore distributive equity across regions; and, fourth, it performs sensitivity analysis under today’s elevated interest rates and inflationary context to explicitly link macro-financial conditions to transition dynamics. We thus suggest that exploring how realistic costs and perceived investment risks may evolve in the future is also critical to understanding how energy systems could shape in response to different financing developments and in pursuit of climate targets: different cost-of-capital developments for different technologies and across regions could mean vastly different winners and losers in the race to climate neutrality—in terms of major (or earlier) shifts in electricity and energy systems, stickiness and sensitivity of certain technologies to financing conditions, infrastructure lock-ins or stranding, and emissions. For example, increasing risks in fossil fuels would consistently benefit climate goals across all regions, leading to divestments in coal, oil, and gas and lowering CO_2_ emissions globally—and more prominently in high-income and some middle-income economies. Instead, favourable conditions for low-carbon technologies alone, e.g., by policies mitigating or reallocating investment risks, could decrease emissions in Latin America, China, and Africa through renewable energy capacity additions and increased green electrification.

Collectively, the two trends of derisking RES and increased fossil fuel risks perceived by our experts hinted that informing energy planning models with more reliable financing conditions and assumptions would reveal different mitigation pathways with different costs and timelines and better inform policies to increase clean capital allocation^31^. Notably, our study highlights that with fixed mitigation efforts, our expert-informed WACC projections could contribute towards achieving longer-term targets, closing the global NDC-LTT gap by as much as 6%—and variably around the world, with up to three times as much in Africa. This is despite a minor increase in emissions in the West, and notably the US, which could nonetheless mostly be attributed in overreliance on not-readily-available technologies like BECCS, the perceived risk of which might be underestimated in current model-based energy planning^52^. A large gap would however remain, indicating that addressing the gaps between high- and low-income economies in costs of capital alone is not a sufficient incentive to help deliver on Paris Agreement-compatible targets. On the other hand, exacerbated differences in the costs of capital between regions would limit access to climate finance and in turn hamper decarbonisation efforts in LMICs, thereby delaying the achievement of global climate goals.

Policies aimed at improving financing conditions and facilitating access to affordable finance could bridge the cost-of-capital gap between high- and low-income economies and help promote decarbonisation pathways aligned with climate policies and targets. For LMICs, renewable energy offers opportunities for socioeconomic development, increased access to affordable clean energy, and energy security. As such, LMICs have set NDC targets with the intention to double their RES installed capacity by 2030, but more than half of the capacity yet to be added is conditional on international support in the form of financing, technical assistance, and other forms of support^53^. Several studies have assessed the impact of subsidies towards clean energy investments, finding that—when accompanied by strong governance mechanisms—they can help LMICs scale up their electricity access rate^54,55^ and enhance technology diffusion^55^. Windfall tax revenues could thus provide a much-needed short-term financial support, a view also expressed by small island nations during COP27^56^.

Our results indicate that taxing windfall profits generated in 2022 alone and redistributing the revenues in a just manner could lower CO_2_ emissions and electricity prices in the mid-term in LMICs, increase industry and building electrification, and benefit RES investments even in high-income economies. Although such a policy would not disrupt fossil-fuel competitiveness, nor create subsidies adequately high to drive cleantech investments, it could send a robust signal to investors that governments are committed to sustainable energy efforts and enhance investor confidence in such projects that can initiate learning-by-doing processes that in turn can, in the long run, help reduce the cost of capital for such investments. This could also signal to investors and policymakers that a collaborative world, which more actively works towards convergence^23^, is more efficient than a protectionist, diverging world that sees emissions growing and global investment opportunities decreasing.

Green finance instruments and blended finance mechanisms offer concrete pathways for implementing the derisking and capital mobilisation assumed in our scenarios. Instruments such as green bonds, sustainability-linked debt, concessional loans, credit guarantees, and risk-sharing facilities can lower perceived investment risks and reduce financing costs, particularly in middle- and high-income markets. For instance, green bonds have mobilised over USD 2 trillion globally since 2014, with dedicated sovereign issuances in countries such as Nigeria, Chile, and Indonesia demonstrating how stable policy frameworks can attract international investors to renewable energy projects^57^. Concessional lending and credit guarantees, for example, through the Climate Investment Funds or the Green Climate Fund’s Private Sector Facility, have helped lower the cost of capital for solar and wind projects in Sub-Saharan Africa and South Asia by partially underwriting currency and political risks^58^. Blended finance, which combines public and private capital to improve risk–return profiles, has proven especially effective in scaling up climate and nature investments^59–61^. Notable examples include the Sustainable Energy for Africa (SEFA) Fund^62^, Denmark’s Investment Fund for Developing Countries (IFU)^63^, and the EU’s EFSD+ guarantee mechanism^64^, all of which crowd in private capital through first-loss tranches or concessional co-financing. Credit enhancement tools and sustainability-linked loans have similarly gained traction, where debt repayment terms are tied to renewable deployment or emissions performance targets^65^. Strengthening these mechanisms, alongside regulatory innovation by central banks, and providing incentives such as subsidies or tax breaks could encourage investments in sustainable energy infrastructure^66^, and help align real-world financial systems with the cost of capital trajectories explored in our study, thereby improving the feasibility of the transition pathways modelled.

Despite increasing efforts to take stock of up-to-date, regionally and technologically disaggregated costs of capital, such as those from IRENA, IEA and recent studies^67^, challenges remain in making robust projections, which can in turn have a high impact on assessing low-carbon investment attractiveness. Being able to develop scenarios that represent plausible cost-of-capital projections is paramount to fostering confidence among investors, enhancing the effectiveness of public and private finance, accelerating the global energy transition, and filling a gap for the broader energy-economic modelling community^68^. Critical infrastructure is under increasing climate change-related pressure, influencing perceived risks associated with certain technologies and regions. To fully capture the benefits of mitigation, it is essential to consider the impact of climate change on the costs of capital by employing more realistic baseline assumptions and trajectories^69^. As the main source of WACC variation lies in each region’s local context and framework conditions, our exercise illustrates the importance of international and regional support alike for the reduction of the assumed cost of capital. This in turn highlights the need for policy and regulatory support towards reducing the real and perceived investment risks, and thus the premiums and costs of capital, as well as providing adequate financial support in LMICs.

However, it is important to acknowledge that project development in LMICs face additional barriers of deployment that go beyond the risks described in the paper. Enabling infrastructure, human capital, institutional and organisational capacity, and availability of data and information, among other factors, often make it hard to turn capital into actual capacity additions, even when capital is available at concessional rates. Although it is possible to execute successful clean energy projects in LMICs, the speed and scale of deployment implied by IAM results overlook the constraints of community acceptance and permitting challenges. Moreover, the present study does not assess the distributional implications of choosing this policy strategy—i.e., redistributing windfall profits to LMICs to boost clean energy deployment—over other strategies, such as domestically offsetting high energy costs among vulnerable households in higher-income countries—which was largely implemented in the light of the energy crisis.

A broader shortcoming in most state-of-the-art IAMs, including GCAM, is that they treat capital markets largely via exogenous cost of capital parameters, ignoring the endogenous responses of financial institutions^70^. While our sensitivity analysis is intended to account for changes in the global risk-free rate, interest rates in IAMs remain exogenous and thus do not reflect the potential adaptive role of central banks in influencing macro-financial conditions. Beyond setting benchmark rates, central banks are progressively integrating climate risks into their mandates and instruments, including green collateral frameworks, differentiated refinancing operations, and disclosure requirements that steer capital allocation. Such measures can indirectly influence the cost of capital and the perceived risk profile of clean energy investments^71,72^. Incorporating these adaptive monetary policy responses into IAMs would allow interest rates and WACC values to evolve endogenously in response to inflation, energy shocks, and climate-related financial risks, thereby capturing two-way feedback between financial regulation, investor expectations, and technology diffusion^73^.

## Methods

### Expert elicitation

Expert inclusion in climate-economy modelling has been proven useful to maximise real-world feasibility of model insights from a societal perspective, and to improve model accuracy and relevance with inputs informed by stakeholder consultations^74^. Here, we use a policy response mechanism developed in an EU-funded research project (IAM COMPACT^75^) and turn to experts from academia, policy and financial institutions, and international organisations to elicit their knowledge and perceptions in the form of a survey^76^ on how interest rates might develop in the future and, in turn, to co-design our study pathways.

Experts were selected through a targeted purposive sampling approach to ensure representation of diverse institutional perspectives, professional backgrounds, and areas of expertise relevant to energy system investment, finance, and macroeconomic policy. The panel comprised experts from European institutions who collectively engage in both EU and non-EU regional analysis and policy work. In order to bring in non-EU perspectives, we included experts with relevant non-EU expertise but cannot rule out that the lack of non-EU-based experts may introduce biases, which is a limitation of our study that follow-up work could tackle. Participants represented a broad range of institutional types, including central and national banks (e.g., the European Central Bank— ECB, National Bank of Belgium), European and national policy institutions (e.g., the European Commission’s Directorate-General for Economic and Financial Affairs—DG ECFIN, Danish Ministry of Finance), international organisations (e.g., Organisation for Economic Co-operation and Development—OECD, World Resources Institute—WRI, European Bank for Reconstruction and Development—EBRD, GIZ), think tanks and NGOs (e.g., Centre for European Policy Studies—CEPS, Third Generation Environmentalism—E3G, Renewables Grid Initiative—RGI, Transport & Environment—T&E, European Association for Electromobility—AVERE), and academia (Utrecht University). Experts were identified and invited based on public records of their expertise or professional responsibilities in the fields of sustainable finance, energy transition modelling, climate risk assessment, and energy decarbonisation. While all experts were affiliated with organisations based in Europe, many of their portfolios and analytical mandates cover non-EU and global regions, ensuring broader geographic relevance.

Specifically, we draw the experts’ opinion on how much investment risks (e.g., risks in power markets, permits, social acceptance, resource and technology, currency, geo-/political) may change for a set of technologies in 2050 in high-, middle-, and low-income countries, considering possible finance and policy derisking or divestment mechanisms that may play out, to determine the final risk percentage change. As a baseline, we provide empirically estimated WACC values from Calcaterra et al.^23^ and IRENA^17^ by technology (coal, natural gas, nuclear, hydro, biomass, solar PV, onshore wind, offshore wind, and green hydrogen) and country-income classification (high, medium, low). To mitigate “groupthink”, expert elicitation followed a two-step process: (i) an individual, anonymised online survey to capture independent judgments; and (ii) in-depth discussions to clarify reasoning behind diverging estimates, without revealing individual responses. Expert input was provided in the form of most-likely values for projected investment risk changes by technology and income group, which were then averaged; baseline WACC values and the resulting experts’ projections are provided in Table S1.

The follow-up discussions focused on the factors that might drive down technology costs at rates faster than previously assumed, and revealed their interest in the role of subsidies in increasing the feasibility of clean energy projects, the expansion of the empirically defined WACC values beyond power technologies—including technologies critical in the decarbonisation of hard-to-abate sectors—as well as the assessment of exploratory pathways of varying interest rates among regions to explore their impacts, without considering solely the cost effectiveness of investments. These discussions, in turn, informed our scenario protocol presented in the “Results” ‘Co-designed projections of costs of capital in the future’.

### GCAM

The Global Change Analysis Model (GCAM)^77^ is an open-source global IAM that represents the behaviour of and interactions between five major systems—energy, land, water, the economy, and climate—at regional and global scales. GCAM is a recursive dynamic simulation model that has been widely used to explore low-carbon policies and mitigation scenarios. GCAM represents economic and energy systems in 32 geopolitical regions and reports demands for and supplies of energy forms as well as emissions of greenhouse gases, aerosols, and other short-lived species^78^.

The model solves for a set of market prices such that supplies and demands are equal for all markets in the model, iterating on these prices until equilibrium is reached. Markets exist for physical flows and other types of goods and services, such as tradable emissions permits. Representative agents interact through the markets, indicating their intended supply and/or demand for goods and services. The current version of GCAM used here (v7) operates from 1990 to 2100 in five-year timesteps, with 2015 as the final calibration year. Once the model solves for each period, it utilises the resulting state of the world, including decisions taken during that time and progresses to the next time step, repeating the process^77^.

### Economic choice

Most of the economic activities represented in GCAM present us with a choice among several ways to produce the result of the activity. The exact share a given option receives in GCAM depends on the logit exponent and the share weight:

1 $${s}_{i}=\frac{{a}_{i}{{c}_{i}}^{\gamma }}{{\sum }_{j=1}^{N}{a}_{j}{{c}_{j}}^{\gamma }}$$

where ${s}_{i}$, ${c}_{i}$, and ${a}_{i}$ are the share, cost, and share weight of technology *i*, respectively, and $\gamma$ is the logit exponent. Logit exponents are exogenously specified and calculated in the historical period, prescribing the degree to which cost, or profit, determines the technology share; exponents that are larger in absolute magnitude result in a more “winner takes all” behaviour^78^. Share weights are used to calibrate the model to observed historical values and allow new technologies to be phased in gradually^23^_._

### Energy demand

Final energy demand includes buildings, transportation, and industry (aluminium, cement, chemicals, construction, fertiliser, iron and steel, mining and agricultural energy use, and other industry) and is driven by exogenous assumptions for income (GDP per capita) and population as well as endogenous changes in the cost of producing a service^79^.

### Energy supply

GCAM models depletable resources (oil, unconventional oil, natural gas, coal, and uranium) using graded resource supply curves. The model’s renewable resources include onshore and offshore wind, solar, geothermal, hydropower, biomass, and in some regions “traditional” biomass; except for biomass, none of these resources are traded between regions. Wind and solar are considered options for producing electricity or hydrogen, while geothermal and hydropower are only used as options for producing electricity. In contrast to the depletable resources, whose cumulative stocks are explicitly tracked, renewable resource quantities are indicated as annual flows.

Hydrogen can be produced from 10 central production technologies (biomass with or without CCS, natural gas steam reforming with and without CCS, thermal splitting, grid electrolysis, dedicated wind and solar electrolysis, nuclear high temperature electrolysis (HTE), and coal chemical with or without CCS) and industrial on-site production technologies (grid electrolysis, renewable electrolysis (green hydrogen), and natural gas with CCS)^79^. Exogenous efficiency assumptions of energy and energy service technologies by region and year capture technical change.

### Electricity

Individual technologies compete for market share based on their technological characteristics, cost of inputs and price of outputs. The cost of a technology in any period depends on its exogenously specified non-energy cost, its endogenously calculated fuel cost, and any cost of emissions, as determined by an imposed climate policy:

2 $${c}_{i}=t+{\sum }_{j=1}^{n}{i}_{j}+\mathop{\sum }_{k=1}^{m}{g}_{k}-{\sum }_{l=1}^{o}{v}_{l}$$

where $${c}_{i}$$ is the total cost of technology *i*, *t* ($) is the exogenously specified technology cost, $${i}_{j}$$ is the cost of input j (e.g., a fuel), $${g}_{k}$$ is the GHG value of gas *k*, and $${v}_{l}$$ is the value of secondary output *l*. Costs vary by region, technology, and year^77^. Fuel or electricity costs depend on the specified efficiency of the technology by region and year and the cost of the fuel or electricity. Non-energy costs represent capital, fixed, and variable O&M costs incurred over the lifetime of the equipment, excluding fuel or electricity costs. For electricity technologies, GCAM reads in each of these terms and computes the levelised cost of energy (LCOE) within the model:

3 $${{{{\rm{LCOE}}}}}_{i}=\frac{{{{{\rm{FCR}}}}}_{i}\cdot {{{{\rm{CAPEX}}}}}_{i}+{{{{\rm{FOM}}}}}_{i}}{{{{{\rm{CF}}}}}_{i}\cdot 8760(\frac{{{{\rm{hours}}}}}{{{{\rm{year}}}}})}+{{{{\rm{VOM}}}}}_{i}$$

where ${{{{\rm{FCR}}}}}_{i}$ is the fixed charge rate of technology *i*, which is the amount of revenue per dollar of investment required that must be collected annually by an investor to pay the carrying charges on that investment, ${{{{\rm{CAPEX}}}}}_{i}$ is capital expenditures to achieve commercial operation, ${{{{\rm{FOM}}}}}_{i}$ the fixed operation and maintenance expenses, ${{{{\rm{CF}}}}}_{i}$ the capacity factor, and ${{{{\rm{VOM}}}}}_{i}$ the variable operation and maintenance^80^.

### Cost of capital

**Fig. 5:**
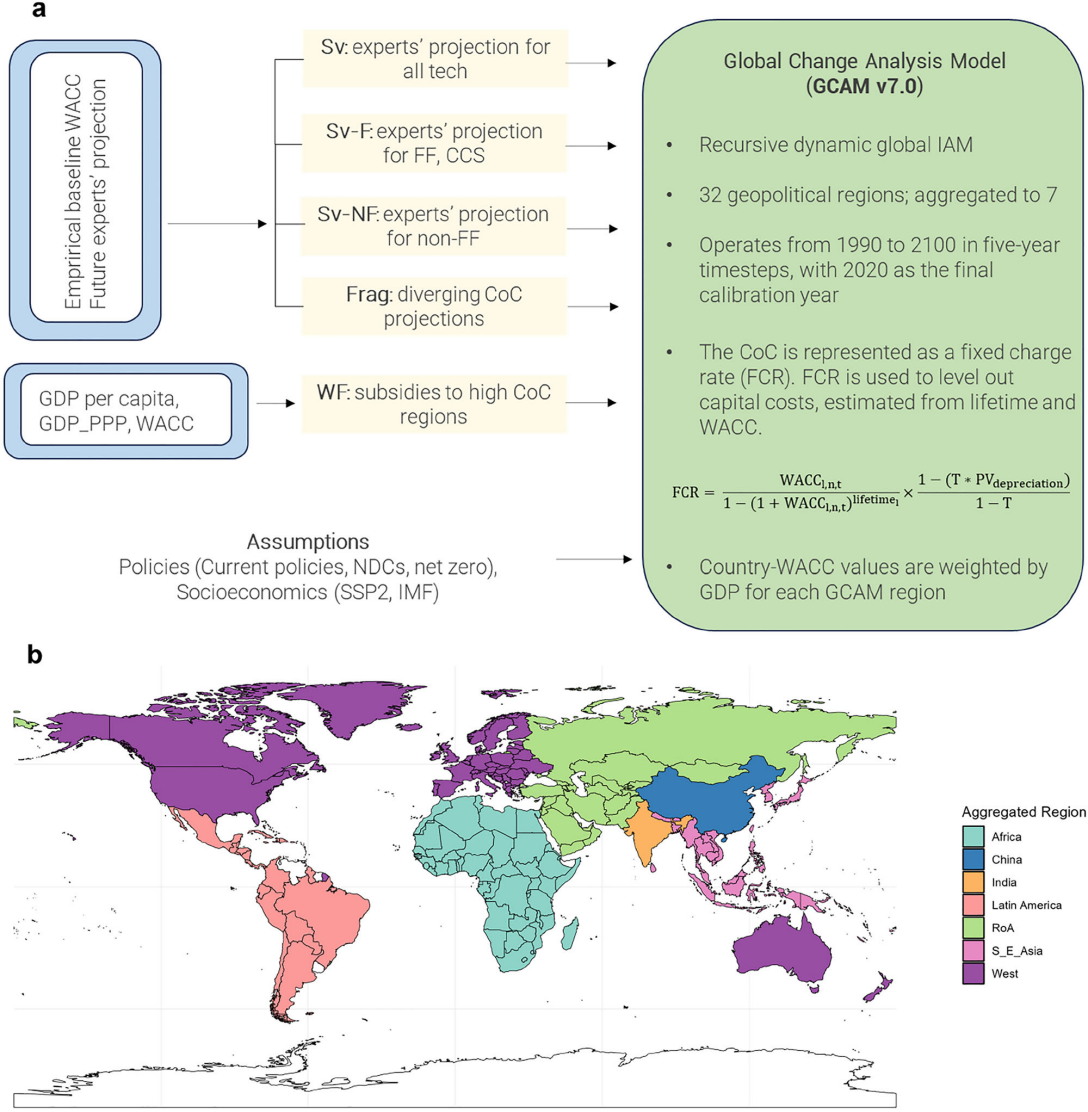
Methodological flow and regional disaggregation of the study. **a** Flow chart visualising inputs, scenarios, and main attributes of the GCAM model that are relevant to this research; **b** regional grouping of countries around the world for the purposes of this research (see Table S2 for the mapping among the study’s macro-regions, GCAM’s geopolitical regions and individual countries). CoC Cost of Capital, FF Fossil Fuels, GDP Gross Domestic Product, IMF International Monetary Fund, NDCs Nationally Determined Contributions, PPP Purchasing Power Parity, PV Present Value, SSP2 Shared Socioeconomic Pathway 2, T Marginal Income Tax Rate, WACC Weighted Average Cost of Capital. Scenarios are explained in Table 1.

The overall cost of capital is derived from a weighted average of all capital sources (debt and equity), known as the weighted average cost of capital (WACC). The WACC serves as the discount rate, indicating that the value of a cash flow depends on the time at which the flow occurs. As the FCR represents all the fixed charges as an annual percentage of the original costs, it levelises the cost of capital^81^. Here, we vary FCR across technologies, regions, and time:

4 $${{{{\rm{FCR}}}}}_{i}=\frac{{{{{\rm{WACC}}}}}_{i,n,t}}{1-{(1+{{{{\rm{WACC}}}}}_{i,n,t})}^{{{{{\rm{lifetime}}}}}_{i}}}\times \frac{1-\left(T *{{{{\rm{PV}}}}}_{{{{\rm{depreciation}}}}}\right)}{1-T}$$

where ${{{{\rm{WACC}}}}}_{i,n,t}$ is the weighted average cost of capital of technology *i*, region $n$, and time $t$; $T$ the marginal income tax rate; ${{{{\rm{lifetime}}}}}_{i}$ the lifetime of technology *i*; and ${{{{\rm{PV}}}}}_{{{{\rm{depreciation}}}}}$ the present value of depreciation^39^. Country-WACC values are weighted by GDP for each aggregated GCAM region (Table S2 and Supplementary Data S1). The methodology is summarised in Fig. 5a, the regional aggregation used in this study is shown in Fig. 5b, while a complete list of countries by Aggregated Region is in Table S2. GCAM does not include endogenous financial experience, which could reduce the WACC values of renewables over time^23^. However, our expert-informed pathways include storylines with decreased WACC values of clean energy technologies, which pushes down financing costs, and makes it easier to generate attractive risk-adjusted returns (Scenarios *Sv, Sv-NF*).

It should be noted that, while WACC is a microeconomic metric typically used in project finance to determine a firm’s required rate of return based on its capital structure and perceived investment risk, GCAM operates at a macroeconomic scale. The model does not simulate firm-level debt–equity ratios, credit conditions, or investor risk behaviour; it instead represents financing costs through FCR, which converts the cost of capital into an annualised payment factor over a technology’s lifetime. In this study, empirically derived and expert-elicited WACC values are used exogenously to inform these FCR parameters by region and technology, thereby translating observed or expected differences in financing conditions into the macroeconomic context of GCAM. This approach bridges the gap between firm-level financial risk and system-wide investment dynamics, allowing us to assess how variations in capital costs can influence regional technology deployment and decarbonisation pathways, without implying that models such as GCAM can directly represent project-specific financial considerations and inform associated decisions.

### Subsidy allocation

Windfall profits from mega-corporations reached 1.1 trillion USD in 2022^29^, which we assumed are taxed by 90%^41^ to provide the total revenue of 990.00 billion USD. For each country *i*, we construct three normalised components: (i) an income-based term proportional to the inverse of GDP per capita, ${E}_{i}$, which favours poorer countries; (ii) an economic-size term based on the country’s share in global GDP at purchasing power parity (PPP), ${G}_{i}$; and (iii) a WACC penalty term, ${N}_{i}$, which is proportional to the country’s WACC when its average RES WACC exceeds a threshold of 6%, and zero otherwise. Countries with ${{\mbox{WACC}}}_{i} < 6\%$ are excluded from the subsidy scheme (their allocation weight is set to zero). For all other countries, we define a raw allocation score, ${\widetilde{s}}_{i}={E}_{i}+{G}_{i}+{N}_{i}$, normalise it, and multiply it by the total subsidy.

Income-based component:

5 $${E}_{i}=\frac{\frac{1}{{{{{\rm{GDP}}}}}_{{{{\rm{pc}}}},i}}}{{\sum }_{j=1}^{N}\frac{1}{{{{{\rm{GDP}}}}}_{{{{\rm{pc}}}},j}}}$$

GDP share component

6 $${G}_{i}=\frac{{{{{\rm{GDP}}}}}_{{{{\rm{PPP}}}},i}}{\mathop{\sum }_{j=1}^{N}{{{{\rm{GDP}}}}}_{{{{\rm{PPP}}}},j}}$$

WACC penalty component:

7 $${P}_{i}={{{\rm{Penalty\; WACC}}}}=\left\{\begin{array}{cc}0,\hfill & {{{\rm{WACC}}}} < 6\%\\ {{{{\rm{WACC}}}}}_{i},& {{{\rm{WACC}}}}\ge 6\%\end{array}\right.$$

8 $${N}_{i}=\frac{{P}_{i}}{\mathop{\sum }_{j=1}^{N}{P}_{j}}$$

Subsidy share:

9 $${\widetilde{s}}_{i}=\left\{\begin{array}{cc}0,\hfill & {{{{\rm{WACC}}}}}_{i} < 6\%\\ {E}_{i}+{G}_{i}+{Ni},& {{{{\rm{WACC}}}}}_{i}\ge 6\%\end{array}\right.$$

Normalised subsidy share:

10 $${s}_{i}=\frac{{\widetilde{s}}_{i}}{\mathop{\sum }_{j=1}^{N}{\widetilde{s}}_{j}}$$

Final subsidy by country:

11 $${{{{\rm{Subsidy}}}}}_{i}={S}_{{{{\rm{total}}}}}\times {s}_{i}$$

where:

${S}_{{{{\rm{total}}}}}$ is the total subsidy budget (i.e., USD_2023_ 990.00 billion), ${{{\rm{GD}}}}{{{{\rm{P}}}}}_{{{{\rm{pc}}}},i}$ is the GDP per capita of country *i*, ${{{{\rm{GDP}}}}}_{{{{\rm{PPP}}}},i}$ is the GDP in purchasing power parity of country *i*, ${{{{\rm{WACC}}}}}_{i}$ is the average WACC value for RES in country *i*, ${P}_{i}$ is the WACC penalty adjustment by country *i* to exclude countries with low-WACC values. The primary purpose of the scheme is to compensate for high financing costs and crowd in private investment, where WACC is a binding constraint. Income and GDP share are kept to ensure equity (favour poorer countries) and scale relevance (larger economies get more absolute support), but the dominant driver is the WACC penalty. Excluding low-WACC countries from the scheme while still using them in the normalisation of ${E}_{i}$ and ${G}_{i}$ ensures that equity and size are benchmarked globally, and not only among eligible recipients. See Table S3 for the allocated regional budget, and Supplementary Data S2 for the detailed subsidy calculation.

We then apply the budget in each region to construct new renewable capacity at the same costs of capital as in the West (Supplementary Data S3), though the model decides how many additional renewables can be built, and the split among technologies. Thus, the degree of subsidisation of each technology also depends on the WACC relative to the West’s WACC, as we compensate the non-West regions’ higher WACC so that the LCOE among all regions is identical (see Supplementary Data S4).

### Reporting summary

Further information on research design is available in the Nature Portfolio Reporting Summary linked to this article.

## Supplementary information

Supplementary Information

Description of Additional Supplementary Files

Supplementary Dataset 1

Supplementary Dataset 2

Supplementary Dataset 3

Supplementary Dataset 4

Supplementary Dataset 5

Supplementary Dataset 6

Reporting Summary

Transparent Peer Review file

## Data Availability

The data (Supplementary Data S1–S6) generated in this study have been deposited in the Zenodo database under accession code 15480070. The research was conducted in accordance with Bruegel’s ethical standards for stakeholder engagement, and found not to meet the threshold requiring review by an Institutional Review Board or equivalent body, also according to the Ethics Research Committee of the National Technical University of Athens (Law 4521/2018): the expert elicitation survey was anonymous and voluntary and involved professional experts only, with no sensitive or personal data collected, and survey data fully anonymised.

## Code availability

The full R code used to produce the figures and the GCAM version used for the modelling runs are publicly available in GitHub^82^ (10.5281/zenodo.19642438).
