# Supplementary material for: Regional impacts on decarbonisation under evolving financing conditions for energy technologies
Source: Nat Commun. 2026 May 19;17:6611. doi: 10.1038/s41467-026-73522-1 (PMC13381915; doi:10.1038/s41467-026-73522-1)
Supplement: Supplementary file 1 — Supplementary Information [file 41467_2026_73522_MOESM1_ESM.pdf]

Supplementary Information

Regional impacts on decarbonisation under evolving financing conditions for energy technologies

Natasha Frilingou*[1], Dirk-Jan Van de Ven[2], Jon Sampedro[2,3], Russell Horowitz[2], Clàudia Rodés-Bachs[2], Thomas Nikolakakis[1], Anastasios Karamaneas[1], Kimon Georgiou[1], Konstantinos Koasidis[1], Shivika Mittal[4], Charalampos Platias[5], Conall Heussaff[6], Christoph Bertram[7,8], Alexandros Nikas*[1]

[1] Energy Policy Unit, School of Electrical & Computer Engineering, National Technical University of Athens, Athens, Greece
[2] Basque Centre for Climate Change (BC3), Leioa, Spain
[3] IKERBASQUE, Basque Foundation for Science, Plaza Euskadi 5, 48009 Bilbao, Spain.
[4] CICERO Center for International Climate Research, Oslo, Norway
[5] Department of International and European Studies, Panteion University of Social & Political Sciences, Athens, Greece
[6] Bruegel, Brussels, Belgium
[7] Center for Global Sustainability (CGS), School of Public Policy, University of Maryland, College Park, MD, USAa
[8] Potsdam Institute for Climate Impact Research (PIK), Member of the Leibniz Association, Potsdam, Germany

* Correspondence: nfrilingou@epu.ntua.gr; anikas@epu.ntua.gr

Table of Contents

Supplementary Figures 3

Figure S1: Regional CO2 emissions trajectories. 3

Figure S2: Global impacts of rising inflation. 3

Figure S3: Regional implications on carbon capture and storage (CCS) of different weighted average cost of capital (WACC) projections on top of Nationally Determined Contributions (NDCs).......... 4

Supplementary Tables 5

Table S1: Baseline (2018), inflated (2024), survey-based (2050), and fragmented (2050) WACC values by country and technology. 5

Table S2: Mapping from macro-regions of the study to GCAM v7.0 geopolitical regions and individual countries. 17

Table S3: Windfall profits tax revenue allocation by Aggregated Region and country 18

Supplementary Figures

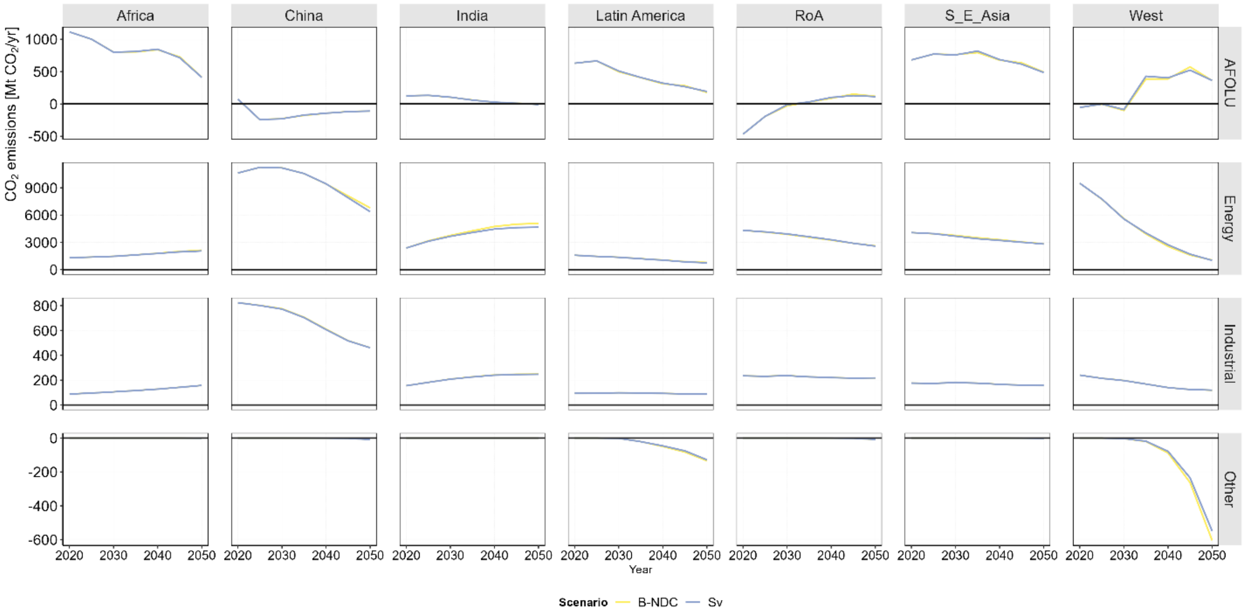

Figure S1: Regional CO2 emissions trajectories. CO_2 emission pathways from agriculture, forestry and other land use (AFOLU), energy, industrial processes, and other (Direct Air Carbon Capture and Storage; DACCS) by region in *Sv* and *B-NDC* scenarios. Scenarios are explained in **Table 1**, while aggregated regions in **Table S2.**

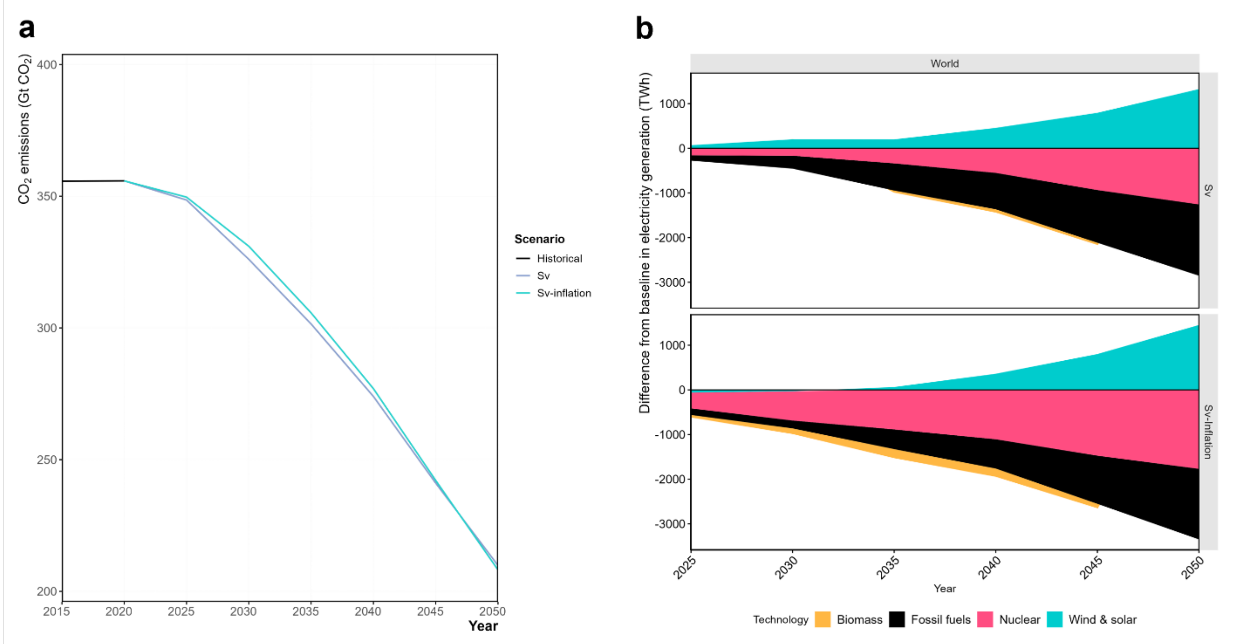

Figure S2: Global impacts of rising inflation. (a) Global CO_2 emissions from energy and industrial processes in scenarios with varying inflation (*Sv* and *Sv-inflation*); (b) Electricity generation by technology relative to the *B-NDC* in scenarios with varying inflation (*Sv* and *Sv-inflation*). Scenarios are explained in **Table 1**.

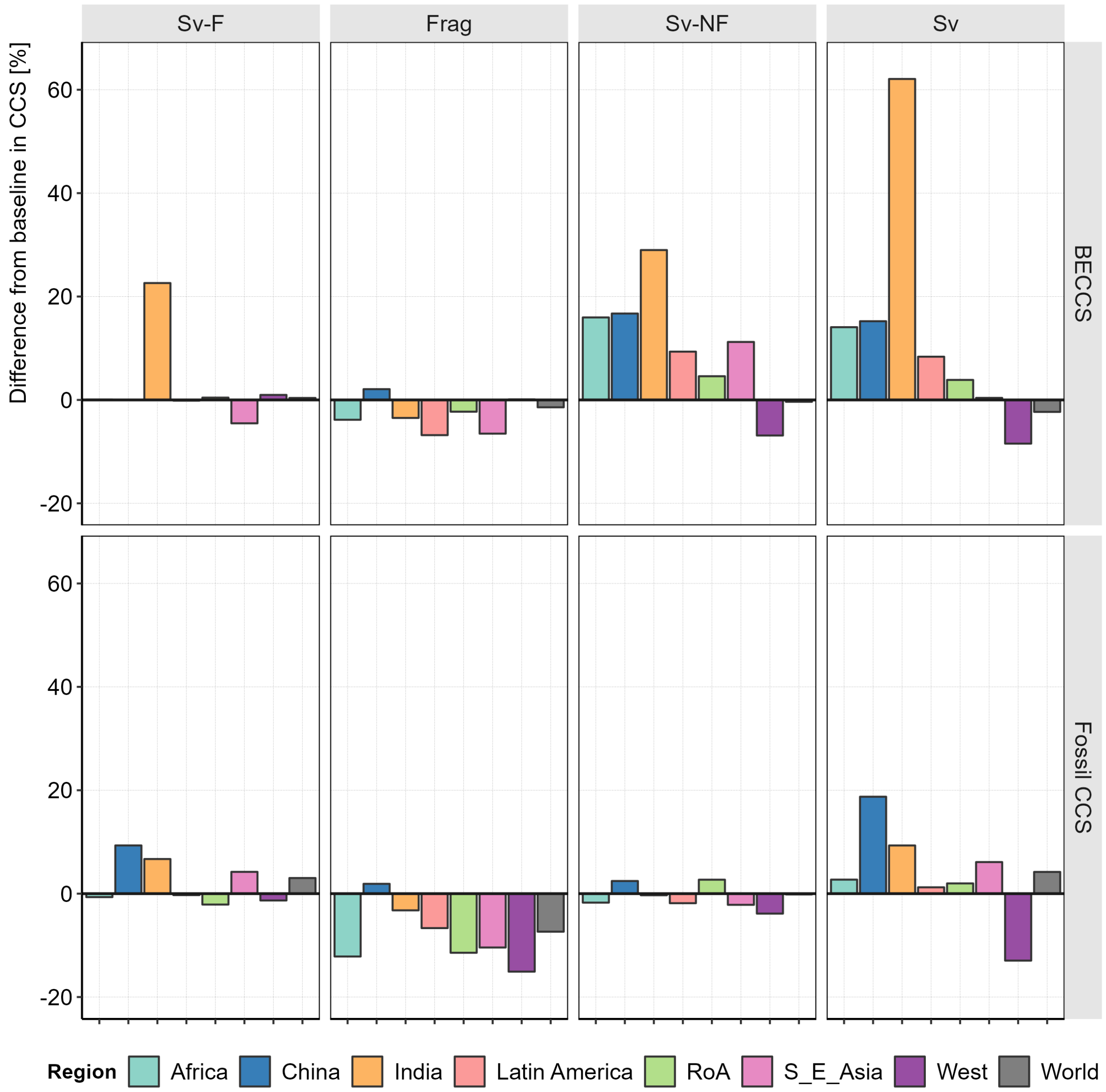

Figure S3: Regional implications on carbon capture and storage (CCS) of different weighted average cost of capital (WACC) projections on top of Nationally Determined Contributions (NDCs). Percentage change of Biomass CCS (BECCS) and Fossil CCS by scenario and region compared to *B-NDC* in 2050.

Supplementary Tables

Table S1: Baseline (2018), inflated (2024), survey-based (2050), and fragmented (2050) weighted average cost of capital (WACC) values by country and technology.

Countries	Year	Coal-fired plant	Gas plants	Nuclear plant	Hydro	Biomass	CCS	Solar PV	Onshore Wind	Offshore Wind	Green Hydrogen
**Baseline WACC values by country and technology taken from (Calcaterra et al., 2024; IRENA, 2022)**											
Algeria	2018	11.4%	9.2%	11.0%	10.2%	11.4%	11.4%	13.1%	13.4%	13.4%	13.3%
Argentina	2018	15.2%	13.0%	14.8%	14.0%	15.2%	15.2%	15.5%	15.8%	15.8%	15.7%
Australia	2018	5.1%	2.9%	4.7%	4.0%	5.1%	5.1%	3.7%	4.8%	6.2%	4.9%
Austria	2018	5.4%	3.2%	5.1%	4.3%	5.4%	5.4%	5.1%	4.3%	6.7%	5.3%
Azerbaijan	2018	7.6%	5.4%	7.3%	6.5%	7.6%	7.6%	8.7%	9.0%	9.0%	8.9%
Bangladesh	2018	8.1%	5.9%	7.8%	7.0%	8.1%	8.1%	8.5%	8.8%	8.8%	8.7%
Belarus	2018	10.5%	8.4%	10.2%	9.4%	10.5%	10.5%	11.6%	11.9%	11.9%	11.8%
Belgium	2018	5.6%	3.4%	5.2%	4.5%	5.6%	5.6%	4.2%	5.4%	5.4%	5.0%
Bolivia	2018	9.7%	7.5%	9.4%	8.6%	9.7%	9.7%	10.4%	10.7%	10.7%	10.6%
Bosnia and Herzegovina	2018	10.5%	8.4%	10.2%	9.4%	10.5%	10.5%	12.1%	12.4%	12.4%	12.3%
Brazil	2018	7.6%	5.4%	7.3%	6.5%	7.6%	7.6%	8.0%	6.8%	8.3%	7.7%
Bulgaria	2018	6.4%	4.2%	6.1%	5.3%	6.4%	6.4%	6.5%	6.8%	8.2%	7.2%
Burkina Faso	2018	9.7%	7.5%	9.4%	8.6%	9.7%	9.7%	7.7%	10.5%	10.5%	9.6%
Canada	2018	5.1%	2.9%	4.7%	4.0%	5.1%	5.1%	6.0%	5.0%	6.3%	5.8%
Chile	2018	5.7%	3.5%	5.3%	4.5%	5.7%	5.7%	5.2%	5.5%	6.8%	5.8%
China	2018	5.7%	3.5%	5.3%	4.5%	5.7%	5.7%	4.4%	4.5%	6.9%	5.3%
Colombia	2018	6.7%	4.5%	6.3%	5.5%	6.7%	6.7%	7.3%	7.6%	7.6%	7.5%
Costa Rica	2018	9.7%	7.5%	9.4%	8.6%	9.7%	9.7%	10.1%	7.6%	10.4%	9.4%
Croatia	2018	7.2%	5.0%	6.8%	6.0%	7.2%	7.2%	8.3%	6.1%	8.6%	7.7%
Cuba	2018	12.6%	10.5%	12.3%	11.5%	12.6%	12.6%	13.3%	13.6%	13.6%	13.5%
Cyprus	2018	7.6%	5.4%	7.3%	6.5%	7.6%	7.6%	7.6%	7.9%	9.3%	8.3%
Czech Republic	2018	5.6%	3.4%	5.2%	4.5%	5.6%	5.6%	5.4%	7.0%	7.0%	6.5%
Denmark	2018	5.1%	2.9%	4.7%	4.0%	5.1%	5.1%	4.8%	4.1%	4.1%	4.3%
Dominican Republic	2018	8.1%	5.9%	7.8%	7.0%	8.1%	8.1%	7.3%	9.0%	9.0%	8.5%

Countries	Year	Coal-fired plant	Gas plants	Nuclear plant	Hydro	Biomass	CCS	Solar PV	Onshore Wind	Offshore Wind	Green Hydrogen
Ecuador	2018	13.5%	11.3%	13.1%	12.3%	13.5%	13.5%	13.9%	14.2%	14.2%	14.1%
Egypt	2018	9.7%	7.5%	9.4%	8.6%	9.7%	9.7%	10.5%	10.8%	10.8%	10.7%
El Salvador	2018	10.5%	8.4%	10.2%	9.4%	10.5%	10.5%	8.2%	11.1%	11.1%	10.1%
Estonia	2018	5.7%	3.5%	5.3%	4.5%	5.7%	5.7%	6.8%	4.7%	7.1%	6.2%
Ethiopia	2018	11.4%	9.2%	11.0%	10.2%	11.4%	11.4%	10.1%	7.7%	10.4%	9.4%
Finland	2018	5.4%	3.2%	5.1%	4.3%	5.4%	5.4%	6.5%	4.4%	6.8%	5.9%
France	2018	5.5%	3.3%	5.2%	4.4%	5.5%	5.5%	5.1%	4.3%	6.7%	5.4%
Germany	2018	5.1%	2.9%	4.7%	4.0%	5.1%	5.1%	3.6%	3.8%	4.8%	4.1%
Ghana	2018	10.5%	8.4%	10.2%	9.4%	10.5%	10.5%	11.2%	11.5%	11.5%	11.4%
Greece	2018	8.1%	5.9%	7.8%	7.0%	8.1%	8.1%	6.4%	6.6%	9.2%	7.4%
Guatemala	2018	7.2%	5.0%	6.8%	6.0%	7.2%	7.2%	8.0%	8.3%	8.3%	8.2%
Honduras	2018	8.9%	6.7%	8.5%	7.7%	8.9%	8.9%	6.5%	7.9%	9.5%	8.0%
Hungary	2018	6.9%	4.7%	6.6%	5.8%	6.9%	6.9%	7.1%	8.8%	8.8%	8.2%
India	2018	6.9%	4.7%	6.6%	5.8%	6.9%	6.9%	6.2%	6.4%	7.9%	6.8%
Indonesia	2018	6.7%	4.5%	6.3%	5.5%	6.7%	6.7%	7.7%	8.0%	8.0%	7.9%
Iran	2018	9.7%	7.5%	9.4%	8.6%	9.7%	9.7%	13.1%	13.4%	13.4%	13.3%
Iraq	2018	11.4%	9.2%	11.0%	10.2%	11.4%	11.4%	11.3%	11.6%	11.6%	11.5%
Ireland	2018	5.8%	3.6%	5.5%	4.7%	5.8%	5.8%	7.2%	5.0%	7.5%	6.6%
Israel	2018	5.7%	3.5%	5.3%	4.5%	5.7%	5.7%	5.4%	7.0%	7.0%	6.4%
Italy	2018	6.9%	4.7%	6.6%	5.8%	6.9%	6.9%	5.4%	6.7%	8.1%	6.8%
Jamaica	2018	9.7%	7.5%	9.4%	8.6%	9.7%	9.7%	8.9%	9.1%	10.7%	9.6%
Japan	2018	5.7%	3.5%	5.3%	4.5%	5.7%	5.7%	4.2%	6.7%	6.7%	5.9%
Jordan	2018	8.9%	6.7%	8.5%	7.7%	8.9%	8.9%	7.5%	8.7%	10.2%	8.8%
Kazakhstan	2018	6.9%	4.7%	6.6%	5.8%	6.9%	6.9%	8.0%	8.3%	8.3%	8.2%
Kenya	2018	9.7%	7.5%	9.4%	8.6%	9.7%	9.7%	10.1%	7.7%	10.4%	9.4%
Korea, Republic of	2018	5.5%	3.3%	5.2%	4.4%	5.5%	5.5%	5.1%	6.8%	6.8%	6.2%
Kuwait	2018	5.7%	3.5%	5.3%	4.5%	5.7%	5.7%	7.0%	7.3%	7.3%	7.2%
Latvia	2018	6.1%	3.9%	5.7%	5.0%	6.1%	6.1%	7.2%	7.5%	7.5%	7.4%
Lebanon	2018	22.9%	20.7%	22.5%	21.7%	22.9%	22.9%	22.7%	23.0%	23.0%	22.9%
Lithuania	2018	5.8%	3.6%	5.5%	4.7%	5.8%	5.8%	7.4%	5.2%	7.7%	6.8%

Countries	Year	Coal-fired plant	Gas plants	Nuclear plant	Hydro	Biomass	CCS	Solar PV	Onshore Wind	Offshore Wind	Green Hydrogen
Luxembourg	2018	5.1%	2.9%	4.7%	4.0%	5.1%	5.1%	4.8%	5.0%	6.4%	5.4%
Malaysia	2018	6.1%	3.9%	5.7%	5.0%	6.1%	6.1%	7.1%	7.3%	7.3%	7.2%
Malta	2018	5.8%	3.6%	5.5%	4.7%	5.8%	5.8%	4.0%	6.7%	6.7%	5.8%
Mauritius	2018	6.7%	4.5%	6.3%	5.5%	6.7%	6.7%	6.3%	8.0%	8.0%	7.5%
Mexico	2018	6.4%	4.2%	6.1%	5.3%	6.4%	6.4%	7.1%	6.0%	7.4%	6.9%
Mongolia	2018	10.5%	8.4%	10.2%	9.4%	10.5%	10.5%	9.7%	8.7%	11.5%	10.0%
Montenegro	2018	8.9%	6.7%	8.5%	7.7%	8.9%	8.9%	10.5%	8.2%	10.8%	9.8%
Morocco	2018	7.2%	5.0%	6.8%	6.0%	7.2%	7.2%	7.8%	5.6%	8.1%	7.1%
Myanmar	2018	15.2%	13.0%	14.8%	14.0%	15.2%	15.2%	11.2%	11.5%	11.5%	11.4%
Namibia	2018	8.1%	5.9%	7.8%	7.0%	8.1%	8.1%	6.1%	8.8%	8.8%	7.9%
Netherlands	2018	5.1%	2.9%	4.7%	4.0%	5.1%	5.1%	3.8%	4.0%	6.4%	4.7%
New Zealand	2018	5.1%	2.9%	4.7%	4.0%	5.1%	5.1%	6.0%	4.9%	6.3%	5.7%
Nicaragua	2018	10.5%	8.4%	10.2%	9.4%	10.5%	10.5%	10.9%	8.3%	11.1%	10.1%
Norway	2018	5.1%	2.9%	4.7%	4.0%	5.1%	5.1%	6.2%	6.5%	6.5%	6.4%
Pakistan	2018	10.5%	8.4%	10.2%	9.4%	10.5%	10.5%	10.9%	11.2%	11.2%	11.1%
Panama	2018	6.7%	4.5%	6.3%	5.5%	6.7%	6.7%	6.0%	6.2%	7.6%	6.6%
Peru	2018	6.1%	3.9%	5.7%	5.0%	6.1%	6.1%	6.9%	7.1%	7.1%	7.0%
Philippines	2018	6.7%	4.5%	6.3%	5.5%	6.7%	6.7%	7.4%	7.7%	7.7%	7.6%
Poland	2018	5.8%	3.6%	5.5%	4.7%	5.8%	5.8%	6.9%	4.8%	7.2%	6.3%
Portugal	2018	6.9%	4.7%	6.6%	5.8%	6.9%	6.9%	8.0%	5.7%	8.3%	7.3%
Romania	2018	6.9%	4.7%	6.6%	5.8%	6.9%	6.9%	6.8%	6.0%	8.5%	7.1%
Russian Federation	2018	6.9%	4.7%	6.6%	5.8%	6.9%	6.9%	8.0%	8.3%	8.3%	8.2%
Rwanda	2018	9.7%	7.5%	9.4%	8.6%	9.7%	9.7%	7.5%	10.4%	10.4%	9.4%
Saudi Arabia	2018	5.7%	3.5%	5.3%	4.5%	5.7%	5.7%	7.9%	8.2%	8.2%	8.1%
Senegal	2018	8.1%	5.9%	7.8%	7.0%	8.1%	8.1%	6.2%	8.9%	8.9%	8.0%
Singapore	2018	5.1%	2.9%	4.7%	4.0%	5.1%	5.1%	6.3%	6.6%	6.6%	6.5%
Slovakia	2018	5.8%	3.6%	5.5%	4.7%	5.8%	5.8%	5.5%	7.2%	7.2%	6.6%
Slovenia	2018	6.1%	3.9%	5.7%	5.0%	6.1%	6.1%	5.9%	7.5%	7.5%	7.0%
South Africa	2018	7.6%	5.4%	7.3%	6.5%	7.6%	7.6%	6.9%	8.6%	8.6%	8.0%
Spain	2018	6.4%	4.2%	6.1%	5.3%	6.4%	6.4%	7.3%	5.1%	7.6%	6.7%

Countries	Year	Coal-fired plant	Gas plants	Nuclear plant	Hydro	Biomass	CCS	Solar PV	Onshore Wind	Offshore Wind	Green Hydrogen
Sri Lanka	2018	11.4%	9.2%	11.0%	10.2%	11.4%	11.4%	12.0%	12.3%	12.3%	12.2%
Sweden	2018	5.1%	2.9%	4.7%	4.0%	5.1%	5.1%	6.2%	4.1%	6.5%	5.6%
Switzerland	2018	5.1%	2.9%	4.7%	4.0%	5.1%	5.1%	4.1%	6.7%	6.7%	5.8%
Taiwan	2018	5.6%	3.4%	5.2%	4.5%	5.6%	5.6%	5.4%	7.0%	7.0%	6.5%
Thailand	2018	6.4%	4.2%	6.1%	5.3%	6.4%	6.4%	6.2%	7.8%	7.8%	7.3%
Tunisia	2018	10.5%	8.4%	10.2%	9.4%	10.5%	10.5%	11.0%	11.3%	11.3%	11.2%
Turkey	2018	9.7%	7.5%	9.4%	8.6%	9.7%	9.7%	9.2%	9.5%	11.0%	9.9%
Uganda	2018	9.7%	7.5%	9.4%	8.6%	9.7%	9.7%	10.1%	10.4%	10.4%	10.3%
Ukraine	2018	10.5%	8.4%	10.2%	9.4%	10.5%	10.5%	11.6%	11.9%	11.9%	11.8%
United Arab Emirates	2018	5.5%	3.3%	5.2%	4.4%	5.5%	5.5%	7.3%	7.6%	7.6%	7.5%
United Kingdom	2018	5.6%	3.4%	5.2%	4.5%	5.6%	5.6%	4.4%	4.6%	4.6%	4.5%
United States of America	2018	5.1%	2.9%	4.7%	4.0%	5.1%	5.1%	6.0%	4.9%	6.3%	5.8%
Uruguay	2018	6.7%	4.5%	6.3%	5.5%	6.7%	6.7%	5.9%	5.1%	7.6%	6.2%
Venezuela	2018	22.9%	20.7%	22.5%	21.7%	22.9%	22.9%	20.4%	20.7%	20.7%	20.6%
Viet Nam	2018	8.1%	5.9%	7.8%	7.0%	8.1%	8.1%	9.1%	9.4%	9.4%	9.3%
Yemen	2018	22.9%	20.7%	22.5%	21.7%	22.9%	22.9%	19.1%	22.6%	22.6%	21.4%
**Inflated WACC values by country and technology**											
Algeria	2024	13.7%	11.5%	13.4%	12.6%	13.7%	13.7%	15.1%	15.4%	15.4%	15.3%
Argentina	2024	17.5%	15.3%	17.1%	16.4%	17.5%	17.5%	17.5%	17.8%	17.8%	17.7%
Australia	2024	7.4%	5.2%	7.1%	6.3%	7.4%	7.4%	5.7%	6.9%	8.3%	7.0%
Austria	2024	7.8%	5.6%	7.4%	6.6%	7.8%	7.8%	7.1%	6.3%	8.7%	7.4%
Azerbaijan	2024	9.9%	7.8%	9.6%	8.8%	9.9%	9.9%	10.7%	11.0%	11.0%	10.9%
Bangladesh	2024	10.4%	8.3%	10.1%	9.3%	10.4%	10.4%	10.6%	10.9%	10.9%	10.8%
Belarus	2024	12.9%	10.7%	12.5%	11.7%	12.9%	12.9%	13.7%	14.0%	14.0%	13.9%
Belgium	2024	7.9%	5.7%	7.6%	6.8%	7.9%	7.9%	6.3%	7.5%	7.5%	7.1%
Bolivia	2024	12.0%	9.9%	11.7%	10.9%	12.0%	12.0%	12.4%	12.7%	12.7%	12.6%
Bosnia and Herzegovina	2024	12.9%	10.7%	12.5%	11.7%	12.9%	12.9%	14.2%	14.5%	14.5%	14.4%
Brazil	2024	9.9%	7.8%	9.6%	8.8%	9.9%	9.9%	10.1%	8.9%	10.4%	9.8%
Bulgaria	2024	8.8%	6.6%	8.4%	7.6%	8.8%	8.8%	8.6%	8.8%	10.2%	9.2%
Burkina Faso	2024	12.0%	9.9%	11.7%	10.9%	12.0%	12.0%	9.7%	12.6%	12.6%	11.6%
Canada	2024	7.4%	5.2%	7.1%	6.3%	7.4%	7.4%	8.1%	7.0%	8.4%	7.8%
Chile	2024	8.0%	5.8%	7.7%	6.9%	8.0%	8.0%	7.3%	7.5%	8.9%	7.9%
China	2024	8.0%	5.8%	7.7%	6.9%	8.0%	8.0%	6.4%	6.6%	9.0%	7.3%

Countries	Year	Coal-fired plant	Gas plants	Nuclear plant	Hydro	Biomass	CCS	Solar PV	Onshore Wind	Offshore Wind	Green Hydrogen
Colombia	2024	9.0%	6.8%	8.7%	7.9%	9.0%	9.0%	9.4%	9.7%	9.7%	9.6%
Costa Rica	2024	12.0%	9.9%	11.7%	10.9%	12.0%	12.0%	12.2%	9.7%	12.4%	11.4%
Croatia	2024	9.5%	7.3%	9.2%	8.4%	9.5%	9.5%	10.4%	8.2%	10.7%	9.7%
Cuba	2024	15.0%	12.8%	14.6%	13.8%	15.0%	15.0%	15.4%	15.7%	15.7%	15.6%
Cyprus	2024	9.9%	7.8%	9.6%	8.8%	9.9%	9.9%	9.7%	9.9%	11.3%	10.3%
Czech Republic	2024	7.9%	5.7%	7.6%	6.8%	7.9%	7.9%	7.5%	9.1%	9.1%	8.5%
Denmark	2024	7.4%	5.2%	7.1%	6.3%	7.4%	7.4%	6.9%	6.1%	6.1%	6.4%
Dominican Republic	2024	10.4%	8.3%	10.1%	9.3%	10.4%	10.4%	9.4%	11.1%	11.1%	10.5%
Ecuador	2024	15.8%	13.6%	15.5%	14.7%	15.8%	15.8%	16.0%	16.3%	16.3%	16.2%
Egypt	2024	12.0%	9.9%	11.7%	10.9%	12.0%	12.0%	12.6%	12.9%	12.9%	12.8%
El Salvador	2024	12.9%	10.7%	12.5%	11.7%	12.9%	12.9%	10.2%	13.2%	13.2%	12.2%
Estonia	2024	8.0%	5.8%	7.7%	6.9%	8.0%	8.0%	8.8%	6.7%	9.1%	8.2%
Ethiopia	2024	13.7%	11.5%	13.4%	12.6%	13.7%	13.7%	12.2%	9.8%	12.4%	11.4%
Finland	2024	7.8%	5.6%	7.4%	6.6%	7.8%	7.8%	8.6%	6.5%	8.9%	8.0%
France	2024	7.8%	5.7%	7.5%	6.7%	7.8%	7.8%	7.1%	6.3%	8.8%	7.4%
Germany	2024	7.4%	5.2%	7.1%	6.3%	7.4%	7.4%	5.7%	5.9%	6.9%	6.1%
Ghana	2024	12.9%	10.7%	12.5%	11.7%	12.9%	12.9%	13.2%	13.5%	13.5%	13.4%
Greece	2024	10.4%	8.3%	10.1%	9.3%	10.4%	10.4%	8.5%	8.7%	11.3%	9.5%
Guatemala	2024	9.5%	7.3%	9.2%	8.4%	9.5%	9.5%	10.1%	10.4%	10.4%	10.3%
Honduras	2024	11.2%	9.0%	10.9%	10.1%	11.2%	11.2%	8.6%	10.0%	11.5%	10.0%
Hungary	2024	9.3%	7.1%	8.9%	8.1%	9.3%	9.3%	9.1%	10.8%	10.8%	10.3%
India	2024	9.3%	7.1%	8.9%	8.1%	9.3%	9.3%	8.3%	8.5%	9.9%	8.9%
Indonesia	2024	9.0%	6.8%	8.7%	7.9%	9.0%	9.0%	9.7%	10.0%	10.0%	9.9%
Iran	2024	12.0%	9.9%	11.7%	10.9%	12.0%	12.0%	15.2%	15.5%	15.5%	15.4%
Iraq	2024	13.7%	11.5%	13.4%	12.6%	13.7%	13.7%	13.3%	13.6%	13.6%	13.5%
Ireland	2024	8.1%	5.9%	7.8%	7.0%	8.1%	8.1%	9.2%	7.1%	9.5%	8.6%
Israel	2024	8.0%	5.8%	7.7%	6.9%	8.0%	8.0%	7.4%	9.0%	9.0%	8.5%
Italy	2024	9.3%	7.1%	8.9%	8.1%	9.3%	9.3%	7.5%	8.7%	10.2%	8.8%
Jamaica	2024	12.0%	9.9%	11.7%	10.9%	12.0%	12.0%	11.0%	11.2%	12.7%	11.6%
Japan	2024	8.0%	5.8%	7.7%	6.9%	8.0%	8.0%	6.2%	8.8%	8.8%	7.9%
Jordan	2024	11.2%	9.0%	10.9%	10.1%	11.2%	11.2%	9.5%	10.7%	12.2%	10.8%
Kazakhstan	2024	9.3%	7.1%	8.9%	8.1%	9.3%	9.3%	10.1%	10.3%	10.3%	10.3%
Kenya	2024	12.0%	9.9%	11.7%	10.9%	12.0%	12.0%	12.2%	9.8%	12.4%	11.4%
Korea, Republic of	2024	7.8%	5.7%	7.5%	6.7%	7.8%	7.8%	7.2%	8.8%	8.8%	8.3%
Kuwait	2024	8.0%	5.8%	7.7%	6.9%	8.0%	8.0%	9.0%	9.3%	9.3%	9.2%
Latvia	2024	8.4%	6.2%	8.1%	7.3%	8.4%	8.4%	9.2%	9.5%	9.5%	9.4%

Countries	Year	Coal-fired plant	Gas plants	Nuclear plant	Hydro	Biomass	CCS	Solar PV	Onshore Wind	Offshore Wind	Green Hydrogen
Lebanon	2024	25.2%	23.0%	24.8%	24.1%	25.2%	25.2%	24.7%	25.0%	25.0%	24.9%
Lithuania	2024	8.1%	5.9%	7.8%	7.0%	8.1%	8.1%	9.4%	7.3%	9.7%	8.8%
Luxembourg	2024	7.4%	5.2%	7.1%	6.3%	7.4%	7.4%	6.8%	7.0%	8.4%	7.4%
Malaysia	2024	8.4%	6.2%	8.1%	7.3%	8.4%	8.4%	9.1%	9.4%	9.4%	9.3%
Malta	2024	8.1%	5.9%	7.8%	7.0%	8.1%	8.1%	6.1%	8.7%	8.7%	7.9%
Mauritius	2024	9.0%	6.8%	8.7%	7.9%	9.0%	9.0%	8.4%	10.1%	10.1%	9.5%
Mexico	2024	8.8%	6.6%	8.4%	7.6%	8.8%	8.8%	9.2%	8.1%	9.5%	8.9%
Mongolia	2024	12.9%	10.7%	12.5%	11.7%	12.9%	12.9%	11.7%	10.8%	13.5%	12.0%
Montenegro	2024	11.2%	9.0%	10.9%	10.1%	11.2%	11.2%	12.5%	10.3%	12.8%	11.9%
Morocco	2024	9.5%	7.3%	9.2%	8.4%	9.5%	9.5%	9.8%	7.6%	10.1%	9.2%
Myanmar	2024	17.5%	15.3%	17.1%	16.4%	17.5%	17.5%	13.2%	13.5%	13.5%	13.4%
Namibia	2024	10.4%	8.3%	10.1%	9.3%	10.4%	10.4%	8.2%	10.9%	10.9%	10.0%
Netherlands	2024	7.4%	5.2%	7.1%	6.3%	7.4%	7.4%	5.8%	6.0%	8.4%	6.8%
New Zealand	2024	7.4%	5.2%	7.1%	6.3%	7.4%	7.4%	8.0%	7.0%	8.3%	7.8%
Nicaragua	2024	12.9%	10.7%	12.5%	11.7%	12.9%	12.9%	12.9%	10.4%	13.2%	12.2%
Norway	2024	7.4%	5.2%	7.1%	6.3%	7.4%	7.4%	8.2%	8.5%	8.5%	8.4%
Pakistan	2024	12.9%	10.7%	12.5%	11.7%	12.9%	12.9%	13.0%	13.3%	13.3%	13.2%
Panama	2024	9.0%	6.8%	8.7%	7.9%	9.0%	9.0%	8.0%	8.2%	9.7%	8.6%
Peru	2024	8.4%	6.2%	8.1%	7.3%	8.4%	8.4%	8.9%	9.2%	9.2%	9.1%
Philippines	2024	9.0%	6.8%	8.7%	7.9%	9.0%	9.0%	9.4%	9.7%	9.7%	9.6%
Poland	2024	8.1%	5.9%	7.8%	7.0%	8.1%	8.1%	9.0%	6.8%	9.3%	8.4%
Portugal	2024	9.3%	7.1%	8.9%	8.1%	9.3%	9.3%	10.0%	7.8%	10.3%	9.4%
Romania	2024	9.3%	7.1%	8.9%	8.1%	9.3%	9.3%	8.8%	8.0%	10.5%	9.1%
Russian Federation	2024	9.3%	7.1%	8.9%	8.1%	9.3%	9.3%	10.1%	10.3%	10.3%	10.3%
Rwanda	2024	12.0%	9.9%	11.7%	10.9%	12.0%	12.0%	9.6%	12.4%	12.4%	11.5%
Saudi Arabia	2024	8.0%	5.8%	7.7%	6.9%	8.0%	8.0%	10.0%	10.3%	10.3%	10.2%
Senegal	2024	10.4%	8.3%	10.1%	9.3%	10.4%	10.4%	8.3%	11.0%	11.0%	10.1%
Singapore	2024	7.4%	5.2%	7.1%	6.3%	7.4%	7.4%	8.4%	8.7%	8.7%	8.6%
Slovakia	2024	8.1%	5.9%	7.8%	7.0%	8.1%	8.1%	7.6%	9.2%	9.2%	8.7%
Slovenia	2024	8.4%	6.2%	8.1%	7.3%	8.4%	8.4%	7.9%	9.6%	9.6%	9.0%
South Africa	2024	9.9%	7.8%	9.6%	8.8%	9.9%	9.9%	8.9%	10.6%	10.6%	10.1%
Spain	2024	8.8%	6.6%	8.4%	7.6%	8.8%	8.8%	9.4%	7.2%	9.7%	8.7%
Sri Lanka	2024	13.7%	11.5%	13.4%	12.6%	13.7%	13.7%	14.1%	14.4%	14.4%	14.3%
Sweden	2024	7.4%	5.2%	7.1%	6.3%	7.4%	7.4%	8.3%	6.1%	8.5%	7.6%
Switzerland	2024	7.4%	5.2%	7.1%	6.3%	7.4%	7.4%	6.1%	8.7%	8.7%	7.9%
Taiwan	2024	7.9%	5.7%	7.6%	6.8%	7.9%	7.9%	7.4%	9.1%	9.1%	8.5%

Countries	Year	Coal-fired plant	Gas plants	Nuclear plant	Hydro	Biomass	CCS	Solar PV	Onshore Wind	Offshore Wind	Green Hydrogen
Thailand	2024	8.8%	6.6%	8.4%	7.6%	8.8%	8.8%	8.2%	9.9%	9.9%	9.3%
Tunisia	2024	12.9%	10.7%	12.5%	11.7%	12.9%	12.9%	13.0%	13.3%	13.3%	13.2%
Turkey	2024	12.0%	9.9%	11.7%	10.9%	12.0%	12.0%	11.3%	11.5%	13.0%	11.9%
Uganda	2024	12.0%	9.9%	11.7%	10.9%	12.0%	12.0%	12.2%	12.4%	12.4%	12.3%
Ukraine	2024	12.9%	10.7%	12.5%	11.7%	12.9%	12.9%	13.7%	14.0%	14.0%	13.9%
United Arab Emirates	2024	7.8%	5.7%	7.5%	6.7%	7.8%	7.8%	9.3%	9.6%	9.6%	9.5%
United Kingdom	2024	7.9%	5.7%	7.6%	6.8%	7.9%	7.9%	6.5%	6.7%	6.7%	6.6%
United States of America	2024	7.4%	5.2%	7.1%	6.3%	7.4%	7.4%	8.1%	7.0%	8.4%	8.3%
Uruguay	2024	9.0%	6.8%	8.7%	7.9%	9.0%	9.0%	8.0%	7.1%	9.7%	7.8%
Venezuela	2024	25.2%	23.0%	24.8%	24.1%	25.2%	25.2%	22.5%	22.7%	22.7%	22.6%
Viet Nam	2024	10.4%	8.3%	10.1%	9.3%	10.4%	10.4%	11.2%	11.5%	11.5%	11.4%
Yemen	2024	25.2%	23.0%	24.8%	24.1%	25.2%	25.2%	21.1%	24.6%	24.6%	23.5%
**WACC values for 2050 by country and technology according to experts' projections**											
Algeria	2050	20%	17%	15%	11%	13%	10%	11%	12%	12%	12%
Argentina	2050	24%	21%	19%	15%	17%	14%	13%	15%	14%	14%
Australia	2050	15%	12%	10%	5%	8%	5%	2%	5%	5%	4%
Austria	2050	15%	12%	10%	5%	8%	5%	4%	5%	5%	5%
Azerbaijan	2050	16%	13%	12%	7%	9%	7%	6%	8%	8%	7%
Bangladesh	2050	17%	14%	12%	8%	10%	7%	6%	8%	7%	7%
Belarus	2050	19%	16%	14%	10%	12%	10%	9%	11%	11%	10%
Belgium	2050	16%	12%	11%	5%	8%	5%	6%	7%	7%	6%
Bolivia	2050	19%	15%	14%	9%	12%	9%	8%	10%	9%	9%
Bosnia and Herzegovina	2050	19%	16%	14%	10%	12%	10%	10%	11%	11%	11%
Brazil	2050	16%	13%	12%	7%	9%	7%	6%	6%	7%	6%
Bulgaria	2050	15%	12%	10%	6%	8%	6%	4%	6%	7%	6%
Burkina Faso	2050	14%	12%	13%	9%	12%	9%	4%	9%	9%	8%
Canada	2050	15%	12%	10%	5%	8%	5%	4%	5%	5%	5%
Chile	2050	16%	12%	11%	5%	8%	5%	3%	4%	4%	3%
China	2050	14%	11%	10%	5%	7%	5%	2%	4%	6%	4%
Colombia	2050	16%	12%	11%	6%	9%	6%	5%	7%	6%	6%
Costa Rica	2050	19%	15%	14%	9%	12%	9%	8%	7%	9%	8%
Croatia	2050	17%	14%	12%	7%	9%	7%	5%	6%	8%	6%
Cuba	2050	21%	18%	17%	12%	14%	12%	11%	13%	12%	12%
Cyprus	2050	17%	14%	12%	7%	10%	7%	7%	6%	8%	7%
Czech Republic	2050	16%	12%	11%	5%	8%	5%	2%	5%	5%	4%
Denmark	2050	15%	12%	10%	5%	8%	5%	3%	4%	3%	3%

Countries	Year	Coal-fired plant	Gas plants	Nuclear plant	Hydro	Biomass	CCS	Solar PV	Onshore Wind	Offshore Wind	Green Hydrogen
Dominican Republic	2050	17%	14%	12%	8%	10%	7%	5%	8%	8%	7%
Ecuador	2050	22%	19%	17%	13%	15%	13%	12%	13%	13%	13%
Egypt	2050	19%	15%	14%	9%	12%	9%	8%	10%	9%	9%
El Salvador	2050	19%	16%	14%	10%	12%	10%	6%	10%	10%	9%
Estonia	2050	16%	12%	11%	5%	8%	5%	3%	5%	6%	5%
Ethiopia	2050	16%	13%	15%	11%	13%	10%	7%	6%	9%	8%
Finland	2050	15%	12%	10%	5%	8%	5%	3%	4%	6%	4%
France	2050	15%	12%	10%	5%	8%	5%	5%	4%	6%	5%
Germany	2050	15%	12%	10%	5%	8%	5%	2%	4%	4%	3%
Ghana	2050	19%	16%	14%	10%	12%	10%	9%	10%	10%	10%
Greece	2050	18%	14%	13%	7%	10%	7%	6%	8%	8%	7%
Guatemala	2050	16%	13%	11%	7%	9%	6%	6%	7%	7%	7%
Honduras	2050	18%	14%	13%	8%	11%	8%	4%	7%	8%	6%
Hungary	2050	17%	13%	12%	6%	9%	6%	4%	5%	7%	5%
India	2050	16%	12%	11%	6%	9%	6%	4%	5%	7%	5%
Indonesia	2050	16%	12%	11%	6%	9%	6%	6%	7%	7%	6%
Iran	2050	19%	15%	14%	9%	12%	9%	11%	12%	12%	12%
Iraq	2050	20%	17%	15%	11%	13%	10%	9%	11%	10%	10%
Ireland	2050	16%	12%	11%	5%	8%	5%	6%	8%	7%	7%
Israel	2050	16%	12%	11%	5%	8%	5%	5%	4%	6%	5%
Italy	2050	17%	14%	12%	7%	9%	7%	5%	9%	8%	7%
Jamaica	2050	19%	15%	14%	9%	12%	9%	7%	8%	9%	8%
Japan	2050	16%	12%	11%	5%	8%	5%	4%	7%	6%	5%
Jordan	2050	18%	14%	13%	8%	11%	8%	5%	8%	9%	7%
Kazakhstan	2050	16%	12%	11%	6%	9%	6%	6%	7%	7%	7%
Kenya	2050	19%	15%	14%	9%	12%	9%	8%	7%	9%	8%
Korea, Republic of	2050	16%	12%	11%	5%	8%	5%	3%	7%	6%	5%
Kuwait	2050	16%	12%	11%	5%	8%	5%	2%	7%	6%	5%
Latvia	2050	16%	12%	11%	6%	8%	6%	4%	7%	6%	5%
Lebanon	2050	32%	28%	27%	22%	25%	22%	21%	22%	22%	21%
Lithuania	2050	16%	12%	11%	6%	8%	6%	5%	5%	7%	5%
Luxembourg	2050	15%	12%	10%	5%	8%	5%	3%	5%	5%	4%
Malaysia	2050	15%	12%	10%	6%	8%	5%	5%	6%	6%	6%
Malta	2050	16%	12%	11%	6%	8%	6%	6%	5%	7%	6%
Mauritius	2050	16%	12%	11%	6%	9%	6%	4%	7%	7%	6%
Mexico	2050	15%	12%	10%	6%	8%	6%	5%	5%	6%	5%

Countries	Year	Coal-fired plant	Gas plants	Nuclear plant	Hydro	Biomass	CCS	Solar PV	Onshore Wind	Offshore Wind	Green Hydrogen
Mongolia	2050	19%	16%	14%	10%	12%	10%	7%	8%	10%	8%
Montenegro	2050	18%	14%	13%	8%	11%	8%	8%	7%	9%	8%
Morocco	2050	16%	13%	11%	7%	9%	6%	6%	5%	7%	6%
Myanmar	2050	24%	21%	19%	15%	17%	14%	9%	10%	10%	10%
Namibia	2050	17%	14%	12%	8%	10%	7%	4%	8%	7%	6%
Netherlands	2050	15%	12%	10%	5%	8%	5%	2%	4%	5%	4%
New Zealand	2050	15%	12%	10%	5%	8%	5%	4%	5%	5%	5%
Nicaragua	2050	19%	16%	14%	10%	12%	10%	9%	7%	10%	9%
Norway	2050	15%	12%	10%	5%	8%	5%	4%	6%	6%	5%
Pakistan	2050	19%	16%	14%	10%	12%	10%	9%	10%	10%	10%
Panama	2050	16%	13%	11%	6%	9%	6%	6%	5%	7%	6%
Peru	2050	15%	12%	10%	6%	8%	5%	5%	6%	6%	6%
Philippines	2050	16%	12%	11%	6%	9%	6%	5%	7%	6%	6%
Poland	2050	16%	12%	11%	6%	8%	6%	2%	7%	6%	5%
Portugal	2050	17%	14%	12%	7%	9%	7%	4%	7%	7%	6%
Romania	2050	17%	14%	12%	7%	9%	7%	6%	6%	7%	6%
Russian Federation	2050	16%	12%	11%	6%	9%	6%	6%	7%	7%	7%
Rwanda	2050	14%	12%	13%	9%	12%	9%	4%	9%	9%	8%
Saudi Arabia	2050	16%	12%	11%	5%	8%	5%	5%	7%	6%	6%
Senegal	2050	17%	14%	12%	8%	10%	7%	4%	8%	8%	6%
Singapore	2050	15%	12%	10%	5%	8%	5%	5%	6%	6%	5%
Slovakia	2050	16%	12%	11%	6%	8%	6%	5%	5%	6%	5%
Slovenia	2050	16%	13%	11%	6%	9%	6%	5%	7%	7%	6%
South Africa	2050	16%	13%	12%	7%	9%	7%	5%	8%	7%	6%
Spain	2050	16%	13%	11%	6%	9%	6%	4%	7%	7%	6%
Sri Lanka	2050	20%	17%	15%	11%	13%	10%	10%	11%	11%	11%
Sweden	2050	15%	12%	10%	5%	8%	5%	4%	4%	6%	4%
Switzerland	2050	15%	12%	10%	5%	8%	5%	2%	6%	6%	5%
Taiwan	2050	16%	12%	11%	5%	8%	5%	4%	7%	6%	5%
Thailand	2050	15%	12%	10%	6%	8%	6%	4%	7%	6%	6%
Tunisia	2050	19%	16%	14%	10%	12%	10%	9%	10%	10%	10%
Turkey	2050	19%	15%	14%	9%	12%	9%	7%	8%	10%	8%
Uganda	2050	14%	12%	13%	9%	12%	9%	7%	9%	9%	9%
Ukraine	2050	19%	16%	14%	10%	12%	10%	9%	11%	11%	10%
United Arab Emirates	2050	16%	12%	11%	5%	8%	5%	3%	4%	6%	4%
United Kingdom	2050	16%	12%	11%	5%	8%	5%	4%	7%	6%	5%

Countries	Year	Coal-fired plant	Gas plants	Nuclear plant	Hydro	Biomass	CCS	Solar PV	Onshore Wind	Offshore Wind	Green Hydrogen
United States of America	2050	18%	15%	13%	8%	11%	8%	5%	6%	8%	6%
Uruguay	2050	17%	13%	12%	6%	9%	6%	4%	6%	7%	5%
Venezuela	2050	32%	28%	27%	22%	25%	22%	18%	20%	19%	19%
Viet Nam	2050	17%	14%	12%	8%	10%	7%	7%	8%	8%	8%
Yemen	2050	27%	25%	26%	22%	25%	22%	16%	21%	21%	20%
**Diverging WACC values for 2050 by country and technology**											
Algeria	2050	10.5%	8.4%	10.2%	9.4%	10.5%	10.5%	11.2%	11.5%	11.5%	11.4%
Argentina	2050	10.5%	8.4%	10.2%	9.4%	10.5%	10.5%	11.2%	11.5%	11.5%	11.4%
Australia	2050	5.1%	2.9%	4.7%	4.0%	5.1%	5.1%	5.1%	4.8%	6.5%	5.7%
Austria	2050	5.1%	2.9%	4.7%	4.0%	5.1%	5.1%	5.1%	4.8%	6.5%	5.7%
Azerbaijan	2050	10.5%	8.4%	10.2%	9.4%	10.5%	10.5%	11.2%	11.5%	11.5%	11.4%
Bangladesh	2050	10.5%	8.4%	10.2%	9.4%	10.5%	10.5%	11.2%	11.5%	11.5%	11.4%
Belarus	2050	10.5%	8.4%	10.2%	9.4%	10.5%	10.5%	11.2%	11.5%	11.5%	11.4%
Belgium	2050	5.1%	2.9%	4.7%	4.0%	5.1%	5.1%	5.1%	4.8%	6.5%	5.7%
Bolivia	2050	10.5%	8.4%	10.2%	9.4%	10.5%	10.5%	11.2%	11.5%	11.5%	11.4%
Bosnia and Herzegovina	2050	10.5%	8.4%	10.2%	9.4%	10.5%	10.5%	11.2%	11.5%	11.5%	11.4%
Brazil	2050	10.5%	8.4%	10.2%	9.4%	10.5%	10.5%	11.2%	11.5%	11.5%	11.4%
Bulgaria	2050	10.5%	8.4%	10.2%	9.4%	10.5%	10.5%	11.2%	11.5%	11.5%	11.4%
Burkina Faso	2050	10.5%	8.4%	10.2%	9.4%	10.5%	10.5%	11.2%	11.5%	11.5%	11.4%
Canada	2050	5.1%	2.9%	4.7%	4.0%	5.1%	5.1%	5.1%	4.8%	6.5%	5.7%
Chile	2050	5.1%	2.9%	4.7%	4.0%	5.1%	5.1%	5.1%	4.8%	6.5%	5.7%
China	2050	5.1%	2.9%	4.7%	4.0%	5.1%	5.1%	5.1%	4.8%	6.5%	5.7%
Colombia	2050	10.5%	8.4%	10.2%	9.4%	10.5%	10.5%	11.2%	11.5%	11.5%	11.4%
Costa Rica	2050	10.5%	8.4%	10.2%	9.4%	10.5%	10.5%	11.2%	11.5%	11.5%	11.4%
Croatia	2050	5.1%	2.9%	4.7%	4.0%	5.1%	5.1%	5.1%	4.8%	6.5%	5.7%
Cuba	2050	10.5%	8.4%	10.2%	9.4%	10.5%	10.5%	11.2%	11.5%	11.5%	11.4%
Cyprus	2050	5.1%	2.9%	4.7%	4.0%	5.1%	5.1%	5.1%	4.8%	6.5%	5.7%
Czech Republic	2050	5.1%	2.9%	4.7%	4.0%	5.1%	5.1%	5.1%	4.8%	6.5%	5.7%
Denmark	2050	5.1%	2.9%	4.7%	4.0%	5.1%	5.1%	5.1%	4.8%	6.5%	5.7%
Dominican Republic	2050	10.5%	8.4%	10.2%	9.4%	10.5%	10.5%	11.2%	11.5%	11.5%	11.4%
Ecuador	2050	10.5%	8.4%	10.2%	9.4%	10.5%	10.5%	11.2%	11.5%	11.5%	11.4%
Egypt	2050	10.5%	8.4%	10.2%	9.4%	10.5%	10.5%	11.2%	11.5%	11.5%	11.4%
El Salvador	2050	10.5%	8.4%	10.2%	9.4%	10.5%	10.5%	11.2%	11.5%	11.5%	11.4%
Estonia	2050	5.1%	2.9%	4.7%	4.0%	5.1%	5.1%	5.1%	4.8%	6.5%	5.7%
Ethiopia	2050	10.5%	8.4%	10.2%	9.4%	10.5%	10.5%	11.2%	11.5%	11.5%	11.4%
Finland	2050	5.1%	2.9%	4.7%	4.0%	5.1%	5.1%	5.1%	4.8%	6.5%	5.7%

Countries	Year	Coal-fired plant	Gas plants	Nuclear plant	Hydro	Biomass	CCS	Solar PV	Onshore Wind	Offshore Wind	Green Hydrogen
France	2050	5.1%	2.9%	4.7%	4.0%	5.1%	5.1%	5.1%	4.8%	6.5%	5.7%
Germany	2050	5.1%	2.9%	4.7%	4.0%	5.1%	5.1%	5.1%	4.8%	6.5%	5.7%
Ghana	2050	10.5%	8.4%	10.2%	9.4%	10.5%	10.5%	11.2%	11.5%	11.5%	11.4%
Greece	2050	5.1%	2.9%	4.7%	4.0%	5.1%	5.1%	5.1%	4.8%	6.5%	5.7%
Guatemala	2050	10.5%	8.4%	10.2%	9.4%	10.5%	10.5%	11.2%	11.5%	11.5%	11.4%
Honduras	2050	10.5%	8.4%	10.2%	9.4%	10.5%	10.5%	11.2%	11.5%	11.5%	11.4%
Hungary	2050	5.1%	2.9%	4.7%	4.0%	5.1%	5.1%	5.1%	4.8%	6.5%	5.7%
India	2050	10.5%	8.4%	10.2%	9.4%	10.5%	10.5%	11.2%	11.5%	11.5%	11.4%
Indonesia	2050	10.5%	8.4%	10.2%	9.4%	10.5%	10.5%	11.2%	11.5%	11.5%	11.4%
Iran	2050	10.5%	8.4%	10.2%	9.4%	10.5%	10.5%	11.2%	11.5%	11.5%	11.4%
Iraq	2050	10.5%	8.4%	10.2%	9.4%	10.5%	10.5%	11.2%	11.5%	11.5%	11.4%
Ireland	2050	5.1%	2.9%	4.7%	4.0%	5.1%	5.1%	5.1%	4.8%	6.5%	5.7%
Israel	2050	5.1%	2.9%	4.7%	4.0%	5.1%	5.1%	5.1%	4.8%	6.5%	5.7%
Italy	2050	5.1%	2.9%	4.7%	4.0%	5.1%	5.1%	5.1%	4.8%	6.5%	5.7%
Jamaica	2050	10.5%	8.4%	10.2%	9.4%	10.5%	10.5%	11.2%	11.5%	11.5%	11.4%
Japan	2050	5.1%	2.9%	4.7%	4.0%	5.1%	5.1%	5.1%	4.8%	6.5%	5.7%
Jordan	2050	10.5%	8.4%	10.2%	9.4%	10.5%	10.5%	11.2%	11.5%	11.5%	11.4%
Kazakhstan	2050	10.5%	8.4%	10.2%	9.4%	10.5%	10.5%	11.2%	11.5%	11.5%	11.4%
Kenya	2050	10.5%	8.4%	10.2%	9.4%	10.5%	10.5%	11.2%	11.5%	11.5%	11.4%
Korea, Republic of	2050	5.1%	2.9%	4.7%	4.0%	5.1%	5.1%	5.1%	4.8%	6.5%	5.7%
Kuwait	2050	5.1%	2.9%	4.7%	4.0%	5.1%	5.1%	5.1%	4.8%	6.5%	5.7%
Latvia	2050	5.1%	2.9%	4.7%	4.0%	5.1%	5.1%	5.1%	4.8%	6.5%	5.7%
Lebanon	2050	10.5%	8.4%	10.2%	9.4%	10.5%	10.5%	11.2%	11.5%	11.5%	11.4%
Lithuania	2050	5.1%	2.9%	4.7%	4.0%	5.1%	5.1%	5.1%	4.8%	6.5%	5.7%
Luxembourg	2050	5.1%	2.9%	4.7%	4.0%	5.1%	5.1%	5.1%	4.8%	6.5%	5.7%
Malaysia	2050	10.5%	8.4%	10.2%	9.4%	10.5%	10.5%	11.2%	11.5%	11.5%	11.4%
Malta	2050	5.1%	2.9%	4.7%	4.0%	5.1%	5.1%	5.1%	4.8%	6.5%	5.7%
Mauritius	2050	10.5%	8.4%	10.2%	9.4%	10.5%	10.5%	11.2%	11.5%	11.5%	11.4%
Mexico	2050	10.5%	8.4%	10.2%	9.4%	10.5%	10.5%	11.2%	11.5%	11.5%	11.4%
Mongolia	2050	10.5%	8.4%	10.2%	9.4%	10.5%	10.5%	11.2%	11.5%	11.5%	11.4%
Montenegro	2050	10.5%	8.4%	10.2%	9.4%	10.5%	10.5%	11.2%	11.5%	11.5%	11.4%
Morocco	2050	10.5%	8.4%	10.2%	9.4%	10.5%	10.5%	11.2%	11.5%	11.5%	11.4%
Myanmar	2050	10.5%	8.4%	10.2%	9.4%	10.5%	10.5%	11.2%	11.5%	11.5%	11.4%
Namibia	2050	10.5%	8.4%	10.2%	9.4%	10.5%	10.5%	11.2%	11.5%	11.5%	11.4%
Netherlands	2050	5.1%	2.9%	4.7%	4.0%	5.1%	5.1%	5.1%	4.8%	6.5%	5.7%
New Zealand	2050	5.1%	2.9%	4.7%	4.0%	5.1%	5.1%	5.1%	4.8%	6.5%	5.7%

Countries	Year	Coal-fired plant	Gas plants	Nuclear plant	Hydro	Biomass	CCS	Solar PV	Onshore Wind	Offshore Wind	Green Hydrogen
Nicaragua	2050	10.5%	8.4%	10.2%	9.4%	10.5%	10.5%	11.2%	11.5%	11.5%	11.4%
Norway	2050	5.1%	2.9%	4.7%	4.0%	5.1%	5.1%	5.1%	4.8%	6.5%	5.7%
Pakistan	2050	10.5%	8.4%	10.2%	9.4%	10.5%	10.5%	11.2%	11.5%	11.5%	11.4%
Panama	2050	5.1%	2.9%	4.7%	4.0%	5.1%	5.1%	5.1%	4.8%	6.5%	5.7%
Peru	2050	10.5%	8.4%	10.2%	9.4%	10.5%	10.5%	11.2%	11.5%	11.5%	11.4%
Philippines	2050	10.5%	8.4%	10.2%	9.4%	10.5%	10.5%	11.2%	11.5%	11.5%	11.4%
Poland	2050	5.1%	2.9%	4.7%	4.0%	5.1%	5.1%	5.1%	4.8%	6.5%	5.7%
Portugal	2050	5.1%	2.9%	4.7%	4.0%	5.1%	5.1%	5.1%	4.8%	6.5%	5.7%
Romania	2050	5.1%	2.9%	4.7%	4.0%	5.1%	5.1%	5.1%	4.8%	6.5%	5.7%
Russian Federation	2050	10.5%	8.4%	10.2%	9.4%	10.5%	10.5%	11.2%	11.5%	11.5%	11.4%
Rwanda	2050	10.5%	8.4%	10.2%	9.4%	10.5%	10.5%	11.2%	11.5%	11.5%	11.4%
Saudi Arabia	2050	5.1%	2.9%	4.7%	4.0%	5.1%	5.1%	5.1%	4.8%	6.5%	5.7%
Senegal	2050	10.5%	8.4%	10.2%	9.4%	10.5%	10.5%	11.2%	11.5%	11.5%	11.4%
Singapore	2050	5.1%	2.9%	4.7%	4.0%	5.1%	5.1%	5.1%	4.8%	6.5%	5.7%
Slovakia	2050	5.1%	2.9%	4.7%	4.0%	5.1%	5.1%	5.1%	4.8%	6.5%	5.7%
Slovenia	2050	5.1%	2.9%	4.7%	4.0%	5.1%	5.1%	5.1%	4.8%	6.5%	5.7%
South Africa	2050	10.5%	8.4%	10.2%	9.4%	10.5%	10.5%	11.2%	11.5%	11.5%	11.4%
Spain	2050	5.1%	2.9%	4.7%	4.0%	5.1%	5.1%	5.1%	4.8%	6.5%	5.7%
Sri Lanka	2050	10.5%	8.4%	10.2%	9.4%	10.5%	10.5%	11.2%	11.5%	11.5%	11.4%
Sweden	2050	5.1%	2.9%	4.7%	4.0%	5.1%	5.1%	5.1%	4.8%	6.5%	5.7%
Switzerland	2050	5.1%	2.9%	4.7%	4.0%	5.1%	5.1%	5.1%	4.8%	6.5%	5.7%
Taiwan	2050	5.1%	2.9%	4.7%	4.0%	5.1%	5.1%	5.1%	4.8%	6.5%	5.7%
Thailand	2050	10.5%	8.4%	10.2%	9.4%	10.5%	10.5%	11.2%	11.5%	11.5%	11.4%
Tunisia	2050	10.5%	8.4%	10.2%	9.4%	10.5%	10.5%	11.2%	11.5%	11.5%	11.4%
Turkey	2050	10.5%	8.4%	10.2%	9.4%	10.5%	10.5%	11.2%	11.5%	11.5%	11.4%
Uganda	2050	10.5%	8.4%	10.2%	9.4%	10.5%	10.5%	11.2%	11.5%	11.5%	11.4%
Ukraine	2050	10.5%	8.4%	10.2%	9.4%	10.5%	10.5%	11.2%	11.5%	11.5%	11.4%
United Arab Emirates	2050	5.1%	2.9%	4.7%	4.0%	5.1%	5.1%	5.1%	4.8%	6.5%	5.7%
United Kingdom	2050	5.1%	2.9%	4.7%	4.0%	5.1%	5.1%	5.1%	4.8%	6.5%	5.7%
United States of America	2050	5.1%	2.9%	4.7%	4.0%	5.1%	5.1%	5.1%	4.8%	6.5%	5.7%
Uruguay	2050	5.1%	2.9%	4.7%	4.0%	5.1%	5.1%	5.1%	4.8%	6.5%	5.7%
Venezuela	2050	10.5%	8.4%	10.2%	9.4%	10.5%	10.5%	11.2%	11.5%	11.5%	11.4%
Viet Nam	2050	10.5%	8.4%	10.2%	9.4%	10.5%	10.5%	11.2%	11.5%	11.5%	11.4%
Yemen	2050	10.5%	8.4%	10.2%	9.4%	10.5%	10.5%	11.2%	11.5%	11.5%	11.4%

Table S2: Mapping from the aggregated regions of the study to GCAM v7.0 geopolitical regions and individual countries.

Aggregated Region	GCAM v7.0 region	Countries
Africa	Africa_Eastern	Algeria, Angola, Benin, Botswana, Burkina Faso, Burundi, Cameroon, Cape Verde, Central African Republic, Chad, Comoros, Congo, Cote d'Ivoire, Democratic Republic of the Congo, Djibouti, Egypt, Equatorial Guinea, Eritrea, Ethiopia, Gabon, Gambia, Ghana, Guinea, Guinea-Bissau, Kenya, Lesotho, Liberia, Libya, Madagascar, Malawi, Mali, Mauritania, Mauritius, Morocco, Mozambique, Namibia, Niger, Nigeria, Reunion, Rwanda, Sao Tome and Principe, Senegal, Sierra Leone, Somalia, South Africa, Sudan, Swaziland, Tanzania, Togo, Tunisia, Uganda, Western Sahara, Zambia, Zimbabwe
	Africa_Northern	
	Africa_Southern	
	Africa_Western	
	South Africa	
China	China	China
India	India	India
Latin America	Argentina	Anguilla, Antigua & Barbuda, Argentina, Aruba, Bahamas, Barbados, Belize, Bermuda, Bolivia, Brazil, Cayman Islands, Chile, Colombia, Costa Rica, Cuba, Dominica, Dominican Republic, Ecuador, El Salvador, French Guiana, Grenada, Guadeloupe, Guatemala, Guyana, Haiti, Honduras, Jamaica, Martinique, Mexico, Montserrat, Netherlands Antilles, Nicaragua, Panama, Paraguay, Peru, Saint Kitts and Nevis, Saint Lucia, Saint Vincent and the Grenadines, Suriname, Trinidad and Tobago, Uruguay, Venezuela
	Brazil	
	Central America and the Caribbean	
	Colombia	
	Mexico	
	South America_Northern	
	South America_Southern	
South and East Asia (S_E_Asia)	Indonesia	Afghanistan, American Samoa, Bangladesh, Bhutan, Brunei Darussalam, Cambodia, Christmas Island, Cocos (Keeling) Islands, Cook Islands, Federated States of Micronesia, Fiji, French Polynesia, Guam, Kiribati, Indonesia, Japan, Lao Peoples Democratic Republic, Malaysia, Maldives, Marshall Islands, Mayotte, Myanmar, Nauru, Nepal, New Caledonia, Niue, Norfolk Island, Northern Mariana Islands, Pacific Islands Trust Territory, Palau, Papua New Guinea, Philippines, Pitcairn Islands, Russian Federation, Turkey, Samoa, Seychelles, Singapore, Solomon Islands, South Korea, Sri Lanka, Taiwan, Thailand, Timor Leste, Tokelau, Tonga, Tuvalu, Vanuatu, Viet Nam
	Japan	
	South Asia	
	Southeast Asia	
	South Korea	
	Taiwan	

Aggregated Region	GCAM v7.0 region	Countries
Rest of Asia (RoA)	Central Asia	Armenia, Azerbaijan, Bahrain, Democratic People's Republic of Korea, Georgia, Iran, Iraq, Israel, Jordan, Kazakhstan, Kuwait, Kyrgyzstan, Lebanon, Mongolia, Oman, Pakistan, Palestine, Qatar, Russian Federation, Saudi Arabia, South Korea, Syria, Tajikistan, Turkmenistan, United Arab Emirates, Uzbekistan, Yemen
	Middle East	
	Pakistan	
	Russia	
West	Australia_NZ	Albania, Andorra, Australia, Austria, Belarus, Belgium, Bosnia and Herzegovina, Bulgaria, Canada, Croatia, Cyprus, Czech Republic, Denmark, Estonia, Finland, France, Germany, Greece, Greenland, Hungary, Iceland, Ireland, Italy, Latvia, Lithuania, Luxembourg, Macedonia, Malta, Moldova, Monaco, Montenegro, Netherlands, New Zealand, Norway, Poland, Portugal, Romania, Serbia, Slovakia, Slovenia, Spain, Sweden, Switzerland, Ukraine, United Kingdom, United States of America
	Canada	
	EU-12	
	EU-15	
	Europe_Eastern	
	European Free Trade Association	
	Europe_Non_EU	
	USA	

Table S3: Windfall profits tax revenue allocation by aggregated region of the study and by country (USD_{2023})

Aggregated Region	Country	Subsidy per country | USD_{2023}
West	Austria	0
West	Belarus	9,890,623,535
West	Belgium	0
West	Bosnia and Herzegovina	10,523,591,899
West	Bulgaria	6,763,516,837
West	Croatia	6,448,458,770
West	Cyprus	5,952,744,430
West	Czech Republic	6,297,191,073
West	Denmark	0
West	Estonia	5,014,245,149
West	Finland	0
West	France	0
West	Germany	0
West	Greece	6,909,847,302
West	Hungary	7,193,783,445
West	Ireland	5,624,597,462
West	Italy	13,559,084,771
West	Latvia	5,975,315,991
West	Lithuania	5,506,086,352
West	Luxembourg	0
West	Malta	0

Aggregated Region	Country	Subsidy per country | USD_{2023}
West	Montenegro	7,953,852,802
West	Netherlands	0
West	Norway	5,466,292,248
West	Poland	9,168,367,912
West	Portugal	6,727,274,256
West	Romania	7,572,010,106
West	Slovakia	5,827,519,300
West	Slovenia	5,454,836,369
West	Spain	11,328,194,730
West	Sweden	0
West	Switzerland	0
West	Ukraine	12,591,800,146
West	United Kingdom	0
West	USA	0
West	Canada	0
West	New Zealand	0
West	Australia	0
Africa	Ethiopia	29,454,523,327
Africa	Egypt	14,287,596,263
Africa	South Africa	10,697,895,460
Africa	Kenya	16,973,861,202
Africa	Uganda	27,389,438,080
Africa	Algeria	13,286,093,306
Africa	Morocco	10,992,665,792
Africa	Ghana	16,440,383,637
Africa	Burkina Faso	29,152,694,807
Africa	Senegal	19,155,442,354
Africa	Rwanda	29,200,577,148
Africa	Tunisia	11,144,957,011
Africa	Namibia	9,291,894,830
Africa	Mauritius	6,459,557,472
Rest of Asia (RoA)	Lebanon	15,942,201,130
Rest of Asia (RoA)	Yemen	36,766,790,396
Rest of Asia (RoA)	Iran	15,040,992,300
Rest of Asia (RoA)	Iraq	12,505,549,126
Rest of Asia (RoA)	Türkiye	15,188,904,708
Rest of Asia (RoA)	Azerbaijan	8,964,482,203
Rest of Asia (RoA)	Kazakhstan	8,111,463,120
Rest of Asia (RoA)	Russian Federation	19,665,855,274
Rest of Asia (RoA)	Saudi Arabia	11,193,348,241
Rest of Asia (RoA)	United Arab Emirates	13,152,833,030
Rest of Asia (RoA)	Kuwait	8,812,587,781
Rest of Asia (RoA)	Israel	8,571,948,309

Aggregated Region	Country	Subsidy per country | USD_{2023}
Rest of Asia (RoA)	Jordan	13,414,473,406
South and East Asia (S_E_Asia)	Sri Lanka	11,410,348,291
South and East Asia (S_E_Asia)	Myanmar	17,985,296,843
South and East Asia (S_E_Asia)	Pakistan	19,604,307,901
South and East Asia (S_E_Asia)	Mongolia	9,753,467,319
South and East Asia (S_E_Asia)	Bangladesh	16,873,138,596
South and East Asia (S_E_Asia)	Indonesia	18,994,329,240
South and East Asia (S_E_Asia)	Philippines	10,340,446,128
South and East Asia (S_E_Asia)	Malaysia	7,069,452,164
South and East Asia (S_E_Asia)	Singapore	5,697,785,035
South and East Asia (S_E_Asia)	India	6,021,516,219
South and East Asia (S_E_Asia)	Thailand	41,204,635,692
South and East Asia (S_E_Asia)	Taiwan	11,091,292,513
South and East Asia (S_E_Asia)	Republic of Korea	5,963,263,422
South and East Asia (S_E_Asia)	China	11,961,705,665
South and East Asia (S_E_Asia)	Japan	0
South and East Asia (S_E_Asia)	Vietnam	0
Latin America	Mexico	14,773,458,779
Latin America	Argentina	14,338,360,311
Latin America	Ecuador	12,764,941,254
Latin America	Nicaragua	14,491,858,145
Latin America	Bolivia	12,046,084,892
Latin America	Costa Rica	8,046,636,698
Latin America	El Salvador	11,467,712,540
Latin America	Guatemala	11,039,641,455
Latin America	Brazil	18,050,916,444
Latin America	Colombia	9,985,439,227
Latin America	Peru	9,115,688,769
Latin America	Honduras	13,465,870,013
Latin America	Panama	5,696,807,306
Latin America	Uruguay	5,871,511,338
Latin America	Chile	0
Latin America	Costa Rica	8,046,636,698
Latin America	Venezuela	17,502,189,990
Latin America	Jamaica	10,317,016,513
