# Supplementary material for: Regional impacts on decarbonisation under evolving financing conditions for energy technologies
Source: Nat Commun. 2026 May 19;17:6611. doi: 10.1038/s41467-026-73522-1 (PMC13381915; doi:10.1038/s41467-026-73522-1)
Supplement: Supplementary file 2 — Description of Additional Supplementary Files [file 41467_2026_73522_MOESM2_ESM.pdf]

Description of Additional Supplementary Files:

Supplementary Data 1: Regional mapping of GCAM

Supplementary Data 2: Allocation of subsidies across countries

Supplementary Data 3: New capacity in terms of renewable energy sources

Supplementary Data 4: Compensation based on countries' Weighted Average Cost of Capital

Supplementary Data 5: Model outputs based on the IAMC template

Supplementary Data 6: Model outputs on electricity prices in GCAM
