# Supplementary material for: Regional impacts on decarbonisation under evolving financing conditions for energy technologies
Source: Nat Commun. 2026 May 19;17:6611. doi: 10.1038/s41467-026-73522-1 (PMC13381915; doi:10.1038/s41467-026-73522-1)
Supplement: Supplementary file 9 — Reporting Summary [file 41467_2026_73522_MOESM9_ESM.pdf]

nature portfolio

Corresponding author(s): Alexandros Nikas

Last updated by author(s): Apr 19, 2026

Reporting Summary

Nature Portfolio wishes to improve the reproducibility of the work that we publish. This form provides structure for consistency and transparency in reporting. For further information on Nature Portfolio policies, see our Editorial Policies and the Editorial Policy Checklist.

Statistics

For all statistical analyses, confirm that the following items are present in the figure legend, table legend, main text, or Methods section.

n/a	Confirmed	
☐	☒	The exact sample size (*n*) for each experimental group/condition, given as a discrete number and unit of measurement
☒	☐	A statement on whether measurements were taken from distinct samples or whether the same sample was measured repeatedly
☒	☐	The statistical test(s) used AND whether they are one- or two-sided<br>*Only common tests should be described solely by name; describe more complex techniques in the Methods section.*
☒	☐	A description of all covariates tested
☒	☐	A description of any assumptions or corrections, such as tests of normality and adjustment for multiple comparisons
☒	☐	A full description of the statistical parameters including central tendency (e.g. means) or other basic estimates (e.g. regression coefficient) AND variation (e.g. standard deviation) or associated estimates of uncertainty (e.g. confidence intervals)
☒	☐	For null hypothesis testing, the test statistic (e.g. *F*, *t*, *r*) with confidence intervals, effect sizes, degrees of freedom and *P* value noted<br>*Give P values as exact values whenever suitable.*
☒	☐	For Bayesian analysis, information on the choice of priors and Markov chain Monte Carlo settings
☒	☐	For hierarchical and complex designs, identification of the appropriate level for tests and full reporting of outcomes
☒	☐	Estimates of effect sizes (e.g. Cohen's *d*, Pearson's *r*), indicating how they were calculated

Our web collection on statistics for biologists contains articles on many of the points above.

Software and code

Policy information about availability of computer code

Data collection	All scenario-specific data input into the models was collected as part of model development. Survey data were collected through an anonymised online questionnaire administered via SurveyMonkey.
Data analysis	Analysis of scenarios as well as all the relevant figure outputs was done through R 4.2.3. The full R code used to produce the figures and the GCAM version (IAM COMPACT version of v-7.0) used for the modelling runs are publicly available in GitHub [Natasha Frilingou. (2026). NatasjaF/Cost_of_capital: Regional impacts on decarbonisation under evolving financing conditions for energy technologies (v1.0.0). Zenodo. https://doi.org/10.5281/zenodo.19642540]

For manuscripts utilizing custom algorithms or software that are central to the research but not yet described in published literature, software must be made available to editors and reviewers. We strongly encourage code deposition in a community repository (e.g. GitHub). See the Nature Portfolio guidelines for submitting code & software for further information.

Data

Policy information about availability of data

All manuscripts must include a data availability statement. This statement should provide the following information, where applicable:

- Accession codes, unique identifiers, or web links for publicly available datasets
- A description of any restrictions on data availability
- For clinical datasets or third party data, please ensure that the statement adheres to our policy

A Data Statement has been included:
The data (Data S1 – S6) generated in this study have been deposited in the Zenodo database under accession code 15480070 (https://doi.org/10.5281/zenodo.15480070). The research was conducted in accordance with Bruegel's ethical standards for stakeholder engagement, and found not to meet the threshold requiring review by an Institutional Review Board or equivalent body, also according to the Ethics Research Committee of the National Technical University of Athens (Law 4521/2018): the expert elicitation survey was anonymous and voluntary and involved professional experts only, with no sensitive or personal data collected, and survey data fully anonymised.

Research involving human participants, their data, or biological material

Policy information about studies with human participants or human data. See also policy information about sex, gender (identity/presentation), and sexual orientation and race, ethnicity and racism.

Reporting on sex and gender	N/A
Reporting on race, ethnicity, or other socially relevant groupings	N/A
Population characteristics	The only characteristics collected from the survey was the professional capacity of participants, namely whether they work on European Policy, National Policy, Financial Institutions, Industry, Research, Civil Society or Other.
Recruitment	Experts were selected through a targeted purposive sampling approach to ensure representation of diverse institutional perspectives, professional backgrounds, and areas of expertise relevant to energy system investment, finance, and macroeconomic policy. The panel comprised experts from European institutions who collectively engage in both EU and non-EU regional analysis and policy work; to bring in non-EU perspectives we included experts with relevant non-EU expertise but cannot rule out that the lack of non-EU based experts may introduces biases
Ethics oversight	The research was conducted in accordance with Bruegel's ethical standards for stakeholder engagement, and found not to meet the threshold requiring review by an Institutional Review Board or equivalent body, also according to the Ethics Research Committee of the National Technical University of Athens (Law 4521/2018): the expert elicitation survey was anonymous and voluntary and involved professional experts only, with no sensitive or personal data collected, and survey data fully anonymised.

Note that full information on the approval of the study protocol must also be provided in the manuscript.

Field-specific reporting

Please select the one below that is the best fit for your research. If you are not sure, read the appropriate sections before making your selection.

☐ Life sciences ☐ Behavioural & social sciences ☒ Ecological, evolutionary & environmental sciences

For a reference copy of the document with all sections, see nature.com/documents/nr-reporting-summary-flat.pdf

Ecological, evolutionary & environmental sciences study design

All studies must disclose on these points even when the disclosure is negative.

Study description	This study examines how region- and technology-specific costs of capital influence global decarbonisation pathways. Using an empirical dataset of financing conditions and expert-informed long-term (de-)risking trends for clean and fossil energy, we quantify their effects on mitigation outcomes and investment patterns. We also evaluate a "corrective justice" policy that taxes corporate windfall profits and reallocates revenues to underwrite low-carbon investments in higher-risk regions.
Research sample	Datasets include those specifying socio-economic conditions, technology characteristics, and historical emissions data, as contained in the (referenced) models used in the study. The existing empirical datasets used in the study are:<br>-Calcaterra, M. et al. Reducing the cost of capital to finance the energy transition in developing countries. Nature Energy 2024 9:10 9, 1241–1251 (2024).<br>-IRENA. Global Hydrogen Trade to Meet the 1.5°C Climate Goal: Green Hydrogen Cost and Potential. Global Hydrogen Trade to Meet the 1.5°C Climate Goal: Green Hydrogen Cost and Potential 1–114 (2022).<br><br>The research sample also includes the responses from 11 experts on projections of the evolution of cost of capital. Experts were selected through a targeted purposive sampling approach to ensure representation of diverse institutional perspectives, professional

	backgrounds, and areas of expertise relevant to energy system investment, finance, and macroeconomic policy. The panel comprised experts from European institutions who collectively engage in both EU and non-EU regional analysis and policy work. In order to bring in non-EU perspectives we included experts with relevant non-EU expertise but cannot rule out that the lack of non-EU based experts may introduces biases, which is a limitation of our study that follow-up work could tackle. Participants represented a broad range of institutional types, including central and national banks (e.g., the European Central Bank – ECB, National Bank of Belgium), European and national policy institutions (e.g., the European Commission's Directorate-General for Economic and Financial Affairs – DG ECFIN, Danish Ministry of Finance), international organisations (e.g., Organisation for Economic Co-operation and Development – OECD, World Resources Institute – WRI, European Bank for Reconstruction and Development – EBRD, GIZ), think tanks and NGOs (e.g., Centre for European Policy Studies – CEPS, Third Generation Environmentalism - E3G, Renewables Grid Initiative – RGI, Transport & Environment – T&E, European Association for Electromobility – AVERE), and academia (Utrecht University). Experts were identified and invited based on public records of their expertise or professional responsibilities in the fields of sustainable finance, energy transition modelling, climate risk assessment, and energy decarbonisation.
Sampling strategy	The scenario choices that constitute the sample for the study were arrived at as described in the study, to explore a wide range of plausible socio-economic, technological and policy conditions. Data sampling was conducted at 5-year intervals.<br><br>Experts were selected through targeted purposive sampling to ensure representation across key institutional types and domains relevant to energy investment, finance, and climate-economy analysis. No formal statistical power calculation or a priori sample size calculation was performed for the expert sample. This is because the purpose of the expert elicitation was not statistical inference to a broader population, but the structured collection of informed judgments to support the development of plausible assumptions on cost-of-capital evolution for use in GCAM. A panel of 11 experts was considered sufficient because it provided coverage across the main institutional domains shaping cost-of-capital expectations, including public finance, monetary policy, international policy, civil society, and academia. Given the specialised nature of the topic and the relatively small pool of individuals with relevant cross-cutting expertise, this sample size is consistent with expert-elicitation and qualitative research designs where depth of knowledge and heterogeneity of viewpoints are more important than large numbers. The resulting sample was therefore judged sufficient to capture a meaningful range of credible perspectives for informing model assumptions, while remaining feasible for in-depth engagement and interpretation.
Data collection	The data were incorporated into the model by a number of project teams over the course of the model's development, with references specified in the manuscript. Survey responses were collected using the online platform SurveyMonkey. The start date was provided to experts and came from other, cited, empirical studies (Calcaterra et al., 2018 / the model year is 2020) and the stop date was 2050. We only collected their long-term assessment of cost of capital trajectories in 2050, and did linear interpolation to determine the cost of capital in each 5-year time step of the model (2025, 2030, 2035, 2040, 2045). In terms of spatial scale, we collected cost of capital projections data in aggregated regions based on income (High-income, Middle-income, and Low-income).
Timing and spatial scale	The model runs from 2015-2100 every 5 years, with associated input data assumptions. The model is global with 32 geopolitical regions, which we aggregate into 7 study regions. The regional aggregation of GCAM and the aggregated regions of the study are described in the Supplementary Information (Table S2).
Data exclusions	No data were excluded from the analysis.
Reproducibility	GCAM is available from an open repository at GitHub, whilst data obtained from other sources are included in the Supplemental Information. The archived version of the model and code used to reproduce the analysis is available in Zenodo. https://doi.org/10.5281/zenodo.19642540
Randomization	Scenarios were grouped according to cost of capital evolution so randomization was not relevant.
Blinding	Blinding was not relevant to this study, which relied on computer simulation using existing data inputs.

Did the study involve field work? ☐ Yes ☒ No

Reporting for specific materials, systems and methods

We require information from authors about some types of materials, experimental systems and methods used in many studies. Here, indicate whether each material, system or method listed is relevant to your study. If you are not sure if a list item applies to your research, read the appropriate section before selecting a response.

Materials & experimental systems

n/a	Involved in the study	
☒	☐	Antibodies
☒	☐	Eukaryotic cell lines
☒	☐	Palaeontology and archaeology
☒	☐	Animals and other organisms
☒	☐	Clinical data
☒	☐	Dual use research of concern
☒	☐	Plants

Methods

n/a	Involved in the study	
☒	☐	ChIP-seq
☒	☐	Flow cytometry
☒	☐	MRI-based neuroimaging

Plants

Seed stocks	N/A
Novel plant genotypes	N/A
Authentication	N/A
