# Supplementary material for: Regional impacts on decarbonisation under evolving financing conditions for energy technologies
Source: Nat Commun. 2026 May 19;17:6611. doi: 10.1038/s41467-026-73522-1 (PMC13381915; doi:10.1038/s41467-026-73522-1)
Supplement: Supplementary file 10 — Transparent Peer Review file [file 41467_2026_73522_MOESM10_ESM.pdf]

nature portfolio

Peer Review File

Regional impacts on decarbonisation under evolving financing conditions for energy technologies

Corresponding Author: Dr Alexandros Nikas

This file contains all reviewer reports in order by version, followed by all author rebuttals in order by version.

Version 0:

Reviewer comments:

Reviewer #1

(Remarks to the Author)
Referee Report
Title: Regional impacts of energy technologies’ cost of capital on decarbonisation
Manuscript ID: NCOMMS-25-39719

Summary
This manuscript investigates how regional and technological variation in the cost of capital —proxied via the Weighted Average Cost of Capital—affects decarbonisation trajectories globally. By integrating empirically grounded WACC values with expert-informed forward projections into the GCAM integrated assessment model, the authors develop several novel scenarios including ones with fossil fuel divestment, renewable energy de-risking, and redistribution of windfall profits from fossil energy firms as subsidies to high-risk regions. The study provides important insights into how financial risk conditions interact with climate policy and affect global emissions outcomes, particularly in emerging and developing economies.

General Assessment
This is a relevant, timely, and well-executed study. It tackles a key blind spot in many integrated assessment modeling efforts—the assumption of uniform WACC across regions and technologies—and convincingly shows how this simplification can obscure meaningful mitigation and policy-relevant dynamics. The manuscript is methodologically rigorous, grounded in an extensive empirical and expert-informed WACC dataset, and presents transparent scenario narratives aligned with current policy frameworks.
The paper offers substantial value to climate finance, energy modeling, and policy communities. The proposed "corrective justice" scenario—redistributing windfall profits to subsidize clean energy in high-risk regions—adds a fresh, policy-relevant contribution.

Comments
1.The expert elicitation process is generally well-described, but more details are needed. For instance, how were the experts selected? Were their backgrounds regionally and sectorally diverse? What measures were taken to mitigate “groupthink” during the workshop? Were uncertainty ranges or confidence intervals reported by experts?

2.In this study, the estimation of the risk-free rate—proxied by the nominal yield on the 10-year US Treasury bond—is updated to reflect current economic conditions (4.018% as of October 2024), and a sensitivity analysis is conducted for the Sv scenario. The intention is to explore the implications of a globally elevated baseline interest rate on the WACC across technologies and regions. However, this approach omits an important macroeconomic actor: central banks. Current IAM frameworks, including our use of GCAM, treat interest rates as exogenous and do not account for the potential adaptive role of monetary policy . Central banks increasingly acknowledge climate risks as relevant to financial stability, and several have introduced green financing incentives, altered collateral frameworks, or begun integrating climate considerations into their policy mandates. Yet, the possible strategic role of central banks in influencing interest rates in response to climate objectives is not modeled here. If possible, incorporate or at least discuss, the endogenous evolution of interest rates and WACC values, influenced by central bank responses to inflation, energy shocks, or climate risks. Such integration would better capture feedback between financial regulation, investor expectations, and technology adoption trajectories, thereby enhancing the realism of transition pathways and policy design.

3.I recommend strengthening and expanding the discussion on green finance instruments, which currently appears only briefly in the Discussion section. Given the paper's emphasis on the role of cost of capital in shaping renewable energy investment, it would be valuable to provide a more comprehensive overview of the tools and channels available to de-risk such investments and/or enhance their returns. This could include mechanisms such as green bonds, blended finance, concessional loans, credit guarantees, sustainability-linked debt instruments, and risk-sharing facilities . Expanding this section would not only enrich the policy relevance of the paper but also provide practical pathways for implementing the modeled scenarios in real-world financial systems.

Notes
On the central banks aspect see for example Campiglio, E., Dafermos, Y., Monnin, P. et al. Climate change challenges for central banks and financial regulators. Nature Clim Change 8, 462–468 (2018); and Shears, E., Meckling, J. & Finnegan, J.J. How central banks address climate and transition risks. Nat Energy 10, 431–432 (2025).

On blended finance solution see for example Grantham Research Institute. “Blended finance for scaling up climate and nature investments (2021); Network for Greening the Financial System. “Scaling up blended finance for climate mitigation and adaptation in EMDEs” (2023); and OECD. “Blended finance guidance for clean energy investment” (2021).

Reviewer #2

(Remarks to the Author)
Review on “Regional impacts of energy technologies’ cost of capital on decarbonization”
General Comments
Developing countries and renewable energy sources in particular face high investment risks that are reflected in a high cost of capital for projects. Managing such costs is thus a key challenge in mobilizing (private) funding for the energy transition in the developing world. The impact of the variation in risk between projects and (to a lesser extent) technologies tends to be captured primarily in the debt share available to projects, followed by the cost of equity and less frequently in the cost of debt which is often relatively standardized within a market. For the cost of debt, differences in the country risk seem to be the primary distinguishing factor. In practice, this means investment decisions are structured around the level of debt that the project risk profile can support from the financiers’ perspective. The target rate of return on equity reflects the risk profile the developer expects and is also influenced by the overall electricity cost the market will support. Clearly a stable regulatory environment helps lower the costs of capital below what it would otherwise be. Thus, the authors address an important issue on how to make renewable energy more widely accessible, particularly to low-income populations. The approach in this study is similar to https://www.nature.com/articles/s41560-024-01606-7
The authors combine the empirical estimates of baseline WACC values with an expert-based approach to produce long-term projections of evolving costs of capital over time. The study further builds on and expands the baseline using a global IAM (GCAM), shifts the focus to regional decarbonization pathways under alternative technological and policy choices, with particular emphasis on clean energy and exploring how divergent WACC futures could emerge from policy choices.
GCAM does not explicitly model LCOE but rather uses a formula that aligns with LCOE calculations to determine the cost of power generation. GCAM models calculate the non-energy costs (capital, fixed O&M) amortized over the plant's life using the capital recovery factor and capacity factor and then add the fuel costs and variable O&M costs, which are dependent on technology efficiency and fuel prices. This approach allows GCAM to assess the financial viability and competitiveness of different energy technologies under various economic and policy scenarios. https://jgcri.github.io/gcam-doc/supply_energy.html#:~:text=For example%2C the non%2Denergy,For more information%2C see electricity
WACC represents the average rate a company pays to its capital providers (debt and equity). It measures the cost of financing, and the minimum return needed on a project to satisfy investors. A higher WACC indicates greater financial risk. It is calculated by weighting the cost of equity and debt based on market value proportions and considering tax benefits on interest expenses. The formula is: WACC = (E/V × Cost of Equity) + (D/V × Cost of Debt × (1-Tax Rate)). Factors affecting WACC include the market values of debt and equity, their costs, and the company's capital structure. The discount rate is used to find the present value of future cash flows in a DCF analysis, reflecting the time value of money and investment risk. Its purpose is to determine if future project cash flows are worth more than the initial investment. WACC is often used as the discount rate in DCF analysis because it represents the overall cost of financing. Factors influencing the discount rate include asset risk, project risks, economic and market conditions, interest rates, and the investment period.
FCR in the context of GCAM refers to the Fixed Charge Rate, a financial parameter representing the annual cost of capital investment for power generation technologies within the energy sector of the model. FCR represents the annual portion of a power plant's capital cost that is paid each year, derived from its expected lifetime and an assumed discount rate. This FCR is used, along with the overnight capital cost and capacity factor, to calculate the levelized cost of energy (LCOE) for different electricity generation technologies, incorporating their non-energy costs. The discount rate reflects the opportunity cost of an investment and the preference for money today rather than in the future. In GCAM, the model uses this rate to annualize the overnight capital cost of power plants. A higher discount rate leads to a higher FCR, while a lower discount rate results in a lower FCR.
FCR in the power sector is an annual cost as a fraction of a project's capital expenditure, designed to cover all fixed costs over an investment's life, including those influenced by the WACC. In essence, the FCR is a specific application of the broader WACC principle to the power sector, translating the overall cost of capital into a fixed annual revenue requirement that must be collected from customers to repay the investment. While WACC is a general measure of a company's or project's cost of raising capital (a blend of debt and equity), FCR is a calculated factor derived from WACC, plus other financial elements like taxes and depreciation, to annualize the total capital cost of a power project. The WACC provides the

fundamental cost of capital for a project, while the Fixed Charge Rate is a specific annual factor derived from that WACC and other variables that is used to determine the required annual revenue to recover a power plant's capital investment.
While both WACC and GCAM model deal with economic concepts, they operate on completely different scales for different purposes:
Scale: WACC is a micro-economic metric used for firm-level financial analysis and investment decisions. GCAM is a macro model that operates on a global or regional scale.
Decision-makers: WACC is used by investors and corporate finance teams. GCAM is used by researchers and policymakers to analyze the effects of climate policy and other large-scale changes.
Application: WACC helps determine if an investment project is financially viable for a single company. GCAM helps explore system-wide trends and policy implications, such as the impact of a carbon tax on the overall energy system and economy.
The title of the manuscript is misleading .
I didn't find any significant progress in comparison to the study that had been published in https://www.nature.com/articles/s41560-024-01606-7. However, multi-model exercises (see https://www.nature.com/articles/s41560-024-01606-7) offer distinct advantages over single-model results which is presented in this study. In addition, I have a question on the method (see my comments below).
Comments
Lines 44-48: "Although climate finance flows overall have been sparing in regions needing them most5, post-COVID debt, high inflation and interest rates, and protectionist policies accompanied by huge subsidies toward domestic industry in major economies—such as the US Inflation Reduction Act (IRA)—have been driving clean energy investments in developing economies further down6."
Reviewer: It is unclear why IRA is driving clean energy investments in developing economies down. The reference 6 can't be found -- https://unctad.org/publication/world-698 investment-report-2023 -- error - page not found.
Lines 306-312: "Subsidies are allocated as a function of three indicators (see Methods): (a) countries' living standards measured as the share of gross domestic product (GDP) per capita for the year 2018 (USDPPP, 2017 per capita) to the total available subsidy, so that countries with lower GDP per capita can get higher subsidies; (b) countries' WACC values for clean energy technologies (solar, wind, green hydrogen), so that countries with very low WACC values—i.e., < 6% (IRENA, 2023)—do not receive subsidies; and (c) countries' capability, measured as a share of countries' GDPPPP to global GDPPPP"
Reviewer: The subsidies allocation methodology is questionable (it is also unclear where these available subsidies came from). For example, the share of gross domestic product (GDP) per capita to the total available subsidy, is the same as a ratio of GDP in country "i" and the total available subsidy multiplied by population in this country. It doesn't make sense to me.
Lines 359-365: "For example, increasing risks in fossil fuels would consistently benefit climate goals across all regions, leading to divestments in coal, oil, and gas and lowering CO2 emissions globally—and more prominently in developed and some rapidly emerging economies. Instead, favorable conditions for low-carbon technologies alone, e.g., by policies mitigating or reallocating investment risks, could decrease emissions in Latin America, China, and Africa through renewable energy capacity additions and increased green electrification."
Reviewer: It is unclear how this study will help derisking RES and increased fossil fuel risks.
Lines 528-520: Subsidy allocation
"Windfall profits from mega-corporations reached 1.1 trillion USD in 2022 29in which we assumed are taxed by revenue68
Reviewer: 29 - Pages not found; 68 - The Internet address (url) you wanted to consult does not exist.
The equation 5 shows that calculation of the share of subsidy by country si doesn't represent the share of anything as a first part of this equation is equal to the following:
(St($)*POPi*POPt)/(GDPi($)*GDPt($))
Where St is total subsidy; POPi is population in country i; POPt is total population; GDPi is GDP in country i ; GDPt is total GDP. Thus, si = 1/$ + % +Pi

Version 1:

Reviewer comments:

Reviewer #1

(Remarks to the Author)
I have now read the revised version of NCOMMS-25-39719A. The authors satisfactorily addressed all of my suggestions and I have no further comments.

(Remarks on code availability)
n.a.

Reviewer #3

(Remarks to the Author)
I am wholly satisfied with the way that the authors have dealt with Reviewer 2's comments. On this basis, I would recommend publication.

(Remarks on code availability)

Open Access This Peer Review File is licensed under a Creative Commons Attribution 4.0 International License, which permits use, sharing, adaptation, distribution and reproduction in any medium or format, as long as you give appropriate credit to the original author(s) and the source, provide a link to the Creative Commons license, and indicate if changes were made.
In cases where reviewers are anonymous, credit should be given to 'Anonymous Referee' and the source.
The images or other third party material in this Peer Review File are included in the article's Creative Commons license, unless indicated otherwise in a credit line to the material. If material is not included in the article's Creative Commons license and your intended use is not permitted by statutory regulation or exceeds the permitted use, you will need to obtain permission directly from the copyright holder.
To view a copy of this license, visit https://creativecommons.org/licenses/by/4.0/

Response to reviewers

Reviewer #1

Reviewer Recommendations	Authors' Responses
**R1.1**<br><br>This manuscript investigates how regional and technological variation in the cost of capital — proxied via the Weighted Average Cost of Capital—affects decarbonisation trajectories globally. By integrating empirically grounded WACC values with expert-informed forward projections into the GCAM integrated assessment model, the authors develop several novel scenarios including ones with fossil fuel divestment, renewable energy de-risking, and redistribution of windfall profits from fossil energy firms as subsidies to high-risk regions. The study provides important insights into how financial risk conditions interact with climate policy and affect global emissions outcomes, particularly in emerging and developing economies.<br><br>This is a relevant, timely, and well-executed study. It tackles a key blind spot in many integrated assessment modeling efforts—the assumption of uniform WACC across regions and technologies—and convincingly shows how this simplification can obscure meaningful mitigation and policy-relevant dynamics. The manuscript is methodologically rigorous, grounded in an extensive empirical and expert-informed WACC dataset, and presents transparent scenario narratives aligned with current policy frameworks.<br>The paper offers substantial value to climate finance, energy modeling, and policy communities. The proposed "corrective justice" scenario—redistributing windfall profits to subsidize clean energy in high-risk regions—adds a fresh, policy-relevant contribution.	We thank the reviewer for their interest in our manuscript and positive remarks. Below, we respond to all points raised, which we found genuinely helpful in improving the quality of our manuscript.
**R1.2**<br><br>The expert elicitation process is generally well-described, but more details are needed. For instance, how were the experts selected? Were their backgrounds regionally and sectorally diverse? What measures were taken to mitigate "groupthink" during the workshop? Were uncertainty ranges or confidence intervals reported by experts?	This is a fair point as we had prioritised reporting on the results of the expert elicitation process. We have now expanded on the description of this process, by adding details on the selection of experts, their backgrounds, the measures we took to mitigate "groupthink" during the workshop, and how responses were reported.<br><br>"*Experts were selected through a targeted purposive sampling approach to ensure representation of diverse institutional*

	perspectives, professional backgrounds, and areas of expertise relevant to energy system investment, finance, and macroeconomic policy. The panel comprised experts from European institutions who collectively engage in both EU and non-EU regional analysis and policy work. In order to bring in non-EU perspectives we included experts with relevant non-EU expertise but cannot rule out that the lack of non-EU based experts may introduces biases, which is a limitation of our study that follow-up work could tackle. Participants represented a broad range of institutional types, including central and national banks (e.g., the European Central Bank – ECB, National Bank of Belgium), European and national policy institutions (e.g., the European Commission's Directorate-General for Economic and Financial Affairs – DG ECFIN, Danish Ministry of Finance), international organisations (e.g., Organisation for Economic Co-operation and Development – OECD, World Resources Institute – WRI, European Bank for Reconstruction and Development – EBRD, GIZ), think tanks and NGOs (e.g., Centre for European Policy Studies – CEPS, Third Generation Environmentalism - E3G, Renewables Grid Initiative – RGI, Transport & Environment – T&E, European Association for Electromobility – AVERE), and academia (Utrecht University). Experts were identified and invited based on public records of their expertise or professional responsibilities in the fields of sustainable finance, energy transition modelling, climate risk assessment, and energy decarbonisation.<br><br>[….]<br><br>*To mitigate "groupthink", expert elicitation followed a two-step process: (i) an individual, anonymised online survey to capture independent judgments; and (ii) in-depth discussions to clarify reasoning behind diverging estimates, without revealing individual responses. Expert input was provided in the form of most-likely values for projected investment risk changes by technology and income group, which were then averaged…"*
**R1.3**<br><br>In this study, the estimation of the risk-free rate—proxied by the nominal yield on the 10-year US Treasury bond—is updated to reflect current	Thank you for this insightful comment highlighting the omission of the role of central banks and the endogeneity of interest rates dynamics. We fully agree that central

economic conditions (4.018% as of October 2024), and a sensitivity analysis is conducted for the Sv scenario. The intention is to explore the implications of a globally elevated baseline interest rate on the WACC across technologies and regions. However, this approach omits an important macroeconomic actor: central banks. Current IAM frameworks, including our use of GCAM, treat interest rates as exogenous and do not account for the potential adaptive role of monetary policy. Central banks increasingly acknowledge climate risks as relevant to financial stability, and several have introduced green financing incentives, altered collateral frameworks, or begun integrating climate considerations into their policy mandates. Yet, the possible strategic role of central banks in influencing interest rates in response to climate objectives is not modeled here. If possible, incorporate or at least discuss, the endogenous evolution of interest rates and WACC values, influenced by central bank responses to inflation, energy shocks, or climate risks. Such integration would better capture feedback between financial regulation, investor expectations, and technology adoption trajectories, thereby enhancing the realism of transition pathways and policy design.	banks are increasingly influential in shaping financial conditions relevant to the low-carbon transition, including through green collateral frameworks, differentiated lending schemes, and integration of climate risk into monetary policy operations. It is also true that, in our study, the risk-free rate—and consequently the WACC—is treated as exogenous, consistent with standard IAM frameworks, including GCAM.<br><br>We have now expanded the discussion to explicitly acknowledge this limitation, by adding the following paragraph:<br><br>*"A broader shortcoming in most state-of-the-art IAMs, including GCAM, is that they treat capital markets largely via exogenous cost of capital parameters, ignoring the endogenous responses of financial institutions[61]. While our sensitivity analysis is intended to account for changes in the global risk-free rate, interest rates in IAMs remain exogenous and thus do not reflect the potential adaptive role of central banks in influencing macro-financial conditions. Beyond setting benchmark rates, central banks are progressively integrating climate risks into their mandates and instruments, including green collateral frameworks, differentiated refinancing operations, and disclosure requirements that steer capital allocation. Such measures can indirectly influence the cost of capital and the perceived risk profile of clean energy investments[62,63]. Incorporating these adaptive monetary policy responses into IAMs would allow interest rates and WACC values to evolve endogenously in response to inflation, energy shocks, and climate-related financial risks, thereby capturing two-way feedbacks between financial regulation, investor expectations, and technology diffusion[64]."*
**R1.4**<br><br>I recommend strengthening and expanding the discussion on green finance instruments, which currently appears only briefly in the Discussion section. Given the paper's emphasis on the role of cost of capital in shaping renewable energy investment, it would be valuable to provide a more comprehensive overview of the tools and channels available to de-risk such investments and/or enhance their returns. This could include mechanisms such as green bonds, blended finance, concessional loans, credit guarantees, sustainability-linked debt instruments, and risk-	Another excellent remark—we fully agree and thank the reviewer for this suggestion on the added value of expanding our discussion with an overview of the tools and channels available to de-risk such investments and/or enhance their returns.<br><br>Aiming indeed to enrich the policy relevance of our paper and provide practical pathways for implementation of our insights in the real world, we have elaborated further on the use of green finance instruments (acknowledging

sharing facilities. Expanding this section would not only enrich the policy relevance of the paper but also provide practical pathways for implementing the modeled scenarios in real-world financial systems.	also the journal's word limitations imposed in this context):<br>*"Green finance instruments and blended finance mechanisms offer concrete pathways for implementing the de-risking and capital mobilisation assumed in our scenarios. Instruments such as green bonds, sustainability-linked debt, concessional loans, credit guarantees, and risk-sharing facilities can lower perceived investment risks and reduce financing costs, particularly in emerging and developing markets. For instance, green bonds have mobilised over USD 2 trillion globally since 2014, with dedicated sovereign issuances in countries such as Nigeria, Chile, and Indonesia demonstrating how stable policy frameworks can attract international investors to renewable energy projects[58]. Concessional lending and credit guarantees, for example through the Climate Investment Funds or the Green Climate Fund's Private Sector Facility, have helped lower the cost of capital for solar and wind projects in Sub-Saharan Africa and South Asia by partially underwriting currency and political risks[59].Blended finance, which combines public and private capital to improve risk–return profiles, has proven especially effective in scaling up climate and nature investments[60–62]. Notable examples include the Sustainable Energy for Africa (SEFA) Fund[63], Denmark's Investment Fund for Developing Countries (IFU)[64], and the EU's EFSD+ guarantee mechanism[65], all of which crowd in private capital through first-loss tranches or concessional co-financing. Credit enhancement tools and sustainability-linked loans have similarly gained traction, where debt repayment terms are tied to renewable deployment or emissions performance targets[66]. Strengthening these mechanisms, alongside regulatory innovation by central banks, and providing incentives such as subsidies or tax breaks could encourage investments in sustainable energy infrastructure[67], and help align real-world financial systems with the cost of capital trajectories explored in our study, thereby improving the feasibility of the transition pathways modelled."*
**Notes**<br>On the central banks aspect see for example Campiglio, E., Dafermos, Y., Monnin, P. et al. Climate change challenges for central banks and	All suggested citations were indeed very helpful and to-the-point and have thus been incorporated in our Discussion (see R1.4).

financial regulators. Nature Clim Change 8, 462–468 (2018); and Shears, E., Meckling, J. & Finnegan, J.J. How central banks address climate and transition risks. Nat Energy 10, 431–432 (2025).<br><br>On blended finance solution see for example Grantham Research Institute. "Blended finance for scaling up climate and nature investments (2021); Network for Greening the Financial System. "Scaling up blended finance for climate mitigation and adaptation in EMDEs" (2023); and OECD. "Blended finance guidance for clean energy investment" (2021).	We would once more like to thank the reviewer for the very valuable feedback and hope that the revisions outlined here and marked up in the manuscript have adequately addressed their comments, which we found instrumental in enhancing our study's clarity and added value.

Reviewer #2

Reviewer Recommendations	Authors' Responses
**R2.1**<br><br>Developing countries and renewable energy sources in particular face high investment risks that are reflected in a high cost of capital for projects. Managing such costs is thus a key challenge in mobilizing (private) funding for the energy transition in the developing world. The impact of the variation in risk between projects and (to a lesser extent) technologies tends to be captured primarily in the debt share available to projects, followed by the cost of equity and less frequently in the cost of debt which is often relatively standardized within a market. For the cost of debt, differences in the country risk seem to be the primary distinguishing factor. In practice, this means investment decisions are structured around the level of debt that the project risk profile can support from the financiers' perspective. The target rate of return on equity reflects the risk profile the developer expects and is also influenced by the overall electricity cost the market will support. Clearly a stable regulatory environment helps lower the costs of capital below what it would otherwise be. Thus, the authors address an important issue on how to make renewable energy more widely accessible, particularly to low-income populations. The approach in this study is similar to https://www.nature.com/articles/s41560-024-01606-7<br>The authors combine the empirical estimates of baseline WACC values with an expert-based approach to produce long-term projections of evolving costs of capital over time. The study further builds on and expands the baseline using a global IAM (GCAM), shifts the focus to regional decarbonization pathways under alternative	First, we would like to thank the reviewer for their time as well as for offering this spot-on summary as well as raising some valuable considerations, starting with highlighting the need to clarify the relationship between microeconomic financial metrics (e.g., WACC), on the one hand, and the GCAM model's macroeconomic representation of investment costs, on the other.<br><br>We agree with the reviewer that the scale of application between WACC and a global IAM such as GCAM is de facto different. However, such models represent detailed processes across sectors/technologies and incorporate microeconomic characteristics of technologies; besides, as operationalised in our study (and the study kindly referred to by the reviewer as well as used as a starting point in our analysis), there are clear ways in which WACC values can be implemented in IAMs and assessed—including in GCAM (see also below).<br><br>We also agree with the reviewer's account of the different application domain and type of decision makers *typically* engaged in WACC and finance analysis, on the one hand, and climate-economy (integrated) assessments, on the other. However, let us emphasise that this is the typical case, in which IAMs such as GCAM are used in the academic and policy worlds to study system-wide trends and implications of climate policies and thus evaluate or undergird and support the design of decarbonisation strategies. Nonetheless, IAMs like GCAM are also frequently used to

technological and policy choices, with particular emphasis on clean energy and exploring how divergent WACC futures could emerge from policy choices.<br><br>GCAM does not explicitly model LCOE but rather uses a formula that aligns with LCOE calculations to determine the cost of power generation. GCAM models calculate the non-energy costs (capital, fixed O&M) amortized over the plant's life using the capital recovery factor and capacity factor and then add the fuel costs and variable O&M costs, which are dependent on technology efficiency and fuel prices. This approach allows GCAM to assess the financial viability and competitiveness of different energy technologies under various economic and policy scenarios. https://jgcri.github.io/gcam-doc/supply_energy.html<br><br>WACC represents the average rate a company pays to its capital providers (debt and equity). It measures the cost of financing, and the minimum return needed on a project to satisfy investors. A higher WACC indicates greater financial risk. It is calculated by weighting the cost of equity and debt based on market value proportions and considering tax benefits on interest expenses. The formula is: WACC = (E/V × Cost of Equity) + (D/V × Cost of Debt × (1-Tax Rate)). Factors affecting WACC include the market values of debt and equity, their costs, and the company's capital structure. The discount rate is used to find the present value of future cash flows in a DCF analysis, reflecting the time value of money and investment risk. Its purpose is to determine if future project cash flows are worth more than the initial investment. WACC is often used as the discount rate in DCF analysis because it represents the overall cost of financing. Factors influencing the discount rate include asset risk, project risks, economic and market conditions, interest rates, and the investment period. FCR in the context of GCAM refers to the Fixed Charge Rate, a financial parameter representing the annual cost of capital investment for power generation technologies within the energy sector of the model. FCR represents the annual portion of a power plant's capital cost that is paid each year, derived from its expected lifetime and an assumed discount rate. This FCR is used, along with the overnight capital cost and capacity factor, to calculate the levelized cost of energy (LCOE) for different electricity generation technologies, incorporating their non-energy costs. The discount rate reflects the opportunity cost of an investment and the preference for money today rather than in	determine the financial viability of large-scale investment projects in the context of climate change mitigation (see e.g., https://doi.org/10.1016/j.egycc.2023.100117)—and increasingly to support financial institutions (including investors, banks, etc.), as for example demonstrated in the growing use of IAMs by the Network for Greening the Financial System (NGFS)—see, e.g., the NGFS long-term and short-term climate scenarios for central banks and supervisors (e.g., https://www.ngfs.net/ngfs-scenarios-portal/glossary/). This is also a gap we attempt to bridge, to the extent possible given the specifications of a model such as GCAM.<br><br>On the one hand, our study does incorporate WACC values, as a proxy of costs of capital of technologies, and uses a global integrated assessment model (GCAM) to assess the implications of empirical WACC values that are projected into the future for mitigation pathways at the regional level. On the other, we also see the reviewer's point. As such, we have revised our title to reflect both the essence of our study and the recommendation raised by the reviewer. The new title now reads *"**Regional impacts on decarbonisation under evolving financing conditions for energy technologies**"*.<br><br>We hope that the change reflects our work more accurately.<br><br>We also share the reviewer's concern in that we need to carefully and thoroughly reflect the difference in scale and applicability between GCAM and WACC values.<br><br>Although we had made an effort to do so in the original version of our manuscript, we have now additionally revised the Methods and Discussion sections to explicitly note that GCAM does not model financing structures or firm-level WACC calculations directly. Instead, we use empirically derived, expert-informed WACC values to parameterise regional differences in the cost of capital through the model's Fixed Charge Rate (FCR) mechanism, which determines the annualised cost of investment for each technology, along the lines suggested by the reviewer. We have also added text to clarify that our study operates at the system level, focusing on how varying regional cost of capital assumptions affect decarbonisation pathways, not on project-level financial performance.

the future. In GCAM, the model uses this rate to annualize the overnight capital cost of power plants. A higher discount rate leads to a higher FCR, while a lower discount rate results in a lower FCR.<br><br>FCR in the power sector is an annual cost as a fraction of a project's capital expenditure, designed to cover all fixed costs over an investment's life, including those influenced by the WACC. In essence, the FCR is a specific application of the broader WACC principle to the power sector, translating the overall cost of capital into a fixed annual revenue requirement that must be collected from customers to repay the investment.<br><br>While WACC is a general measure of a company's or project's cost of raising capital (a blend of debt and equity), FCR is a calculated factor derived from WACC, plus other financial elements like taxes and depreciation, to annualize the total capital cost of a power project.<br><br>The WACC provides the fundamental cost of capital for a project, while the Fixed Charge Rate is a specific annual factor derived from that WACC and other variables that is used to determine the required annual revenue to recover a power plant's capital investment.<br><br>While both WACC and GCAM model deal with economic concepts, they operate on completely different scales for different purposes:<br><br>Scale: WACC is a micro-economic metric used for firm-level financial analysis and investment decisions. GCAM is a macro model that operates on a global or regional scale.<br><br>Decision-makers: WACC is used by investors and corporate finance teams. GCAM is used by researchers and policymakers to analyze the effects of climate policy and other large-scale changes.<br><br>Application: WACC helps determine if an investment project is financially viable for a single company. GCAM helps explore system-wide trends and policy implications, such as the impact of a carbon tax on the overall energy system and economy.<br><br>The title of the manuscript is misleading.	In particular, the 'Cost of capital' subsection in Methods has been revised to include the following disclaimer:<br><br>*"It should be noted that, while WACC is a micro-economic metric typically used in corporate/project finance to determine a firm's required rate of return based on its capital structure and perceived investment risk, GCAM operates at a macro-economic scale. The model does not simulate firm-level debt–equity ratios, credit conditions, or investor risk behaviour; it instead represents financing costs through FCR, which converts the cost of capital into an annualised payment factor over a technology's lifetime. In this study, empirically derived and expert-elicited WACC values are used exogenously to inform these FCR parameters by region and technology, thereby translating observed or expected differences in financing conditions into the macro-economic context of GCAM. This approach bridges the gap between firm-level financial risk and system-wide investment dynamics, allowing us to assess how variations in capital costs can influence regional technology deployment and decarbonisation pathways, without implying that models such as GCAM can directly represent project-specific financial considerations and inform associated decisions."*<br><br>Similarly, the Discussion section has been accordingly revised to include the following caveat:<br><br>"*A broader shortcoming in most state-of-the-art IAMs, including GCAM, is that they treat capital markets largely via exogenous cost of capital parameters, ignoring the endogenous responses of financial institutions*[65] *.While our sensitivity analysis is intended to account for changes in the global risk-free rate, interest rates in IAMs remain exogenous and thus do not reflect the potential adaptive role of central banks in influencing macro-financial conditions. Beyond setting benchmark rates, central banks are progressively integrating climate risks into their mandates and instruments, including green collateral frameworks, differentiated refinancing operations, and disclosure requirements that steer capital allocation. Such measures can indirectly influence the cost of capital and the perceived risk profile of clean energy investments*[66,67]*. Incorporating these adaptive monetary policy responses into IAMs would allow interest rates and WACC values to*

	evolve endogenously in response to inflation, energy shocks, and climate-related financial risks, thereby capturing two-way feedbacks between financial regulation, investor expectations, and technology diffusion[68].”
**R2.2**<br><br>I didn’t find any significant progress in comparison to the study that had been published in https://www.nature.com/articles/s41560-024-01606-7. However, multi-model exercises (see https://www.nature.com/articles/s41560-024-01606-7) offer distinct advantages over single-model results which is presented in this study. In addition, I have a question on the method (see my comments below).	Thank you for this comment. Calcaterra et al. (2024) had provided an important first step in empirically estimating technology- and region-specific WACC values as well as integrating them across a multi-model ensemble to assess their influence on global mitigation costs.<br><br>Our study uses as a starting point and builds specifically on this work by extending their research in several important ways. First, we develop forward-looking, expert-elicited trajectories of the baseline WACC values to explore how financing conditions may evolve over time, rather than assessing static values in time—thereby complementing the realism of modelled pathways, not only in more representative (i.e., empirical) WACC values (as in Calcaterra et al.) but also in realistic (expert-informed) future trajectories of these values. Second, in doing so, we fuse data missing in the database of Calcaterra et al. from other sources (on hydrogen), enhancing the added value of the original dataset. Third, we introduce a new policy experiment, a “corrective justice” subsidy funded through windfall-profit taxation, to additionally test how (alternative) global fiscal instruments could redistribute capital towards higher-risk regions and reinforce (or slow down) transitions around the world. Fourth, we finally embed our analysis in the current macro-financial context of elevated interest rates and inflation, performing sensitivity analysis on the global risk-free rate. We have reflected this in the Discussion section by revising our take on what our study achieves in addition:<br><br>*“Calcaterra et al.[23] recently showed that representing real-world costs of capital across low-carbon technologies and regions is critical to more accurately assess the financing cost of decarbonisation around the world that is implied in mitigation pathways compatible with climate policies and pledges. Our study, in turn, advances the literature in four ways: first, it develops forward-looking, expert-elicited trajectories of the cost of capital rather than using static baseline values; second, in doing so, it expands technology coverage to include hydrogen*

	values and reinforce the added value of the original dataset; third, it embeds financial policy instruments such as risk-underwriting subsidies funded through windfall-profit taxation to explore distributive equity across regions; and, fourth, it performs sensitivity analysis under today's elevated interest rates and inflationary context to explicitly link macro-financial conditions to transition dynamics."<br><br>The fact that some of these innovations would require a larger effort to implement in a multi-model study, we first want to show the usefulness in only one model. This follows past patterns of IAM innovation—as seen, e.g., in ref. [19], which arguably served as a single-model proof of concept to what ref. [23] later expanded upon in a multi-model framework.
**R2.3**<br>Lines 44-48: "Although climate finance flows overall have been sparing in regions needing them most5, post-COVID debt, high inflation and interest rates, and protectionist policies accompanied by huge subsidies toward domestic industry in major economies—such as the US Inflation Reduction Act (IRA)—have been driving clean energy investments in developing economies further down6."<br>Reviewer: It is unclear why IRA is driving clean energy investments in developing economies down. The reference 6 can't be found -- https://unctad.org/publication/world-698<br>investment-report-2023 -- error - page not found.	Apologies, the link initially included was correct, but the PDF version produced by the journal for review purposes must have broken it down into two parts, making it unretrievable. Reference 6 remains (correct):<br><br>https://unctad.org/publication/world-investment-report-2023<br><br>Quoting from the report: *"The number of new measures less favourable to investment remained stable compared with 2021, and their share returned to the pre-pandemic level (28 per cent of non-neutral measures) (figure II.2). In developing countries, less favourable measures constituted only 13 per cent of the total. In contrast, half of the measures adopted by developed countries introduced or reinforced investment restrictions, a decrease of 29 percentage points compared with 2021. Several of these policies were directly or indirectly related to national security concerns about foreign ownership of critical infrastructure, core technologies or other sensitive domestic assets. Often, they represented an extension of restrictions introduced during the pandemic, motivated by the desire to protect sensitive domestic businesses from foreign takeovers (section 2)."*<br><br>We understand the point raised by the reviewer, and have now slightly adjusted this sentence to add more clarity:<br><br>*"Although climate-finance flows overall have been sparing in regions needing them most, post-COVID debt, high inflation and interest rates, and the rise of protectionist industrial*

<table>
<tr><td></td><td>policies in major economies—such as the United States' Inflation Reduction Act (IRA)—have redirected much of the available clean-energy investment toward domestic markets, indirectly constraining capital flows to developing countries[6]."</td></tr>
<tr><td>

R2.4

Lines 306-312: "Subsidies are allocated as a function of three indicators (see Methods): (a) countries' living standards measured as the share of gross domestic product (GDP) per capita for the year 2018 (USDPPP, 2017 per capita) to the total available subsidy, so that countries with lower GDP per capita can get higher subsidies; (b) countries' WACC values for clean energy technologies (solar, wind, green hydrogen), so that countries with very low WACC values—i.e., < 6% (IRENA, 2023)—do not receive subsidies; and (c) countries' capability, measured as a share of countries' GDPPPP to global GDPPPP"

Reviewer: The subsidies allocation methodology is questionable (it is also unclear where these available subsidies came from). For example, the share of gross domestic product (GDP) per capita to the total available subsidy, is the same as a ratio of GDP in country "i" and the total available subsidy multiplied by population in this country. It doesn't make sense to me.

</td><td>

Great point and thank you for this comment, as it provided us with the opportunity to clarify the intended message.

We have thus heavily revised the "Subsidy allocation" subsection in Methods, so that the subsidy allocation is clearer to the reader.

"For each country i, we construct three normalised components: (i) an income-based term proportional to the inverse of GDP per capita, E_i, which favours poorer countries; (ii) an economic-size term based on the country's share in global GDP at purchasing power parity (PPP), G_i; and (iii) a WACC penalty term, N_i, which is proportional to the country's WACC when its average RES WACC exceeds a threshold of 6%, and zero otherwise. Countries with $WACC_i < 6\%$ are excluded from the subsidy scheme (their allocation weight is set to zero). For all other countries, we define a raw allocation score, $\tilde{s}_i = E_i + G_i + N_i$, normalise it, and multiply it with the total subsidy.

Income-based component:

$$E_i = \frac{\frac{1}{GDP_{pc,i}}}{\sum_{j=1}^{N}\frac{1}{GDP_{pc,j}}} \quad (eq. 5)$$

GDP share component:

$$G_i = \frac{GDP_{PPP,i}}{\sum_{j=1}^{N} GDP_{PPP,j}} \quad (eq. 6)$$

WACC penalty component:

$$P_i = Penalty\, WACC = \begin{cases} 0, & WACC < 6\% \\ WACC_i, & WACC \geq 6\% \end{cases} \quad (eq. 7)$$

$$N_i = \frac{P_i}{\sum_{j=1}^{N} P_j} \quad (eq. 8)$$

Subsidy share:

$$\tilde{s}_i = \begin{cases} 0, & WACC_i < 6\% \\ E_i + G_i + Ni, & WACC_i \geq 6\% \end{cases} \quad (eq. 9)$$

Normalised subsidy share:

$$s_i = \frac{\tilde{s}_i}{\sum_{j=1}^{N} \tilde{s}_j} \quad (eq. 10)$$

</td></tr>
</table>

	Final subsidy by country:<br>$$Subsidy_i = S_{total} \times s_i \ (eq.11)$$<br>*where:*<br>*S_{total} is the total subsidy budget (i.e., USD2023 990.00 billion), $GDP_{pc,i}$ is the GDP per capita of country i, $GDP_{PPP,i}$ is the GDP in purchasing power parity of country i, $WACC_i$ is the average WACC value for RES in country I, P_i is the WACC penalty adjustment by country i to exclude countries with low WACC values.*<br>*The primary purpose of the scheme is to compensate for high financing costs and crowd in private investment, where WACC is a binding constraint. Income and GDP share are kept to ensure equity (favour poorer countries) and scale relevance (larger economies get more absolute support), but the dominant driver is the WACC penalty. Excluding low-WACC countries from the scheme while still using them in the normalisation of E_i and G_i ensures that equity and size are benchmarked globally, and not only among eligible recipients. See **Table S3** for the allocated regional budget**,** and **Data S2** for the detailed subsidy calculation."*<br>We have also further clarified that the subsidies stem from taxing 90% of the windfall profits of mega corporations in 2022, amounting to 990 billion USD (2023).<br>*"Windfall profits from mega-corporations reached 1.1 trillion USD in 2022[36] which we assumed are taxed by 90%[69] to provide the total revenue of 990.00 billion USD."*
**R2.5**<br>Lines 359-365: "For example, increasing risks in fossil fuels would consistently benefit climate goals across all regions, leading to divestments in coal, oil, and gas and lowering CO2 emissions globally—and more prominently in developed and some rapidly emerging economies. Instead, favorable conditions for low-carbon technologies alone, e.g., by policies mitigating or reallocating investment risks, could decrease emissions in Latin America, China, and Africa through renewable energy capacity additions and increased green electrification."	First, let us clarify that our study examines the *effects* of de-risking renewable energy sources (RES) and increased fossil-fuel risks on regional energy systems, but it does not itself propose or implement measures to achieve these changes. The analysed mechanisms are in fact represented in the GCAM model through *exogenous variations in the weighted average cost of capital (WACC)*: lower WACC values for clean technologies and higher ones for fossil technologies reflect policy-driven or market-perceived changes in investment risk. These scenario-based WACC adjustments allow us to assess how such financial dynamics could influence

**Reviewer**: It is unclear how this study will help derisking RES and increased fossil fuel risks.	technology deployment and emissions outcomes at the system level.<br><br>Drawing on this valuable remark as well as on a point raised by Reviewer 1, we have significantly expanded our Discussion section on how the risk towards renewable investments can realistically be reduced, based on the findings of our study:<br><br>*"Green finance instruments and blended finance mechanisms offer concrete pathways for implementing the de-risking and capital mobilisation assumed in our scenarios. Instruments such as green bonds, sustainability-linked debt, concessional loans, credit guarantees, and risk-sharing facilities can lower perceived investment risks and reduce financing costs, particularly in emerging and developing markets. For instance, green bonds have mobilised over USD 2 trillion globally since 2014, with dedicated sovereign issuances in countries such as Nigeria, Chile, and Indonesia demonstrating how stable policy frameworks can attract international investors to renewable energy projects[58]. Concessional lending and credit guarantees, for example through the Climate Investment Funds or the Green Climate Fund's Private Sector Facility, have helped lower the cost of capital for solar and wind projects in Sub-Saharan Africa and South Asia by partially underwriting currency and political risks[59].Blended finance, which combines public and private capital to improve risk–return profiles, has proven especially effective in scaling up climate and nature investments[60–62]. Notable examples include the Sustainable Energy for Africa (SEFA) Fund[63], Denmark's Investment Fund for Developing Countries (IFU)[64], and the EU's EFSD+ guarantee mechanism[65], all of which crowd in private capital through first-loss tranches or concessional co-financing. Credit enhancement tools and sustainability-linked loans have similarly gained traction, where debt repayment terms are tied to renewable deployment or emissions performance targets[66]. Strengthening these mechanisms, alongside regulatory innovation by central banks, and providing incentives such as subsidies or tax breaks could encourage investments in sustainable energy infrastructure[67], and help align real-world financial systems with the cost of capital trajectories explored in our study, thereby improving the feasibility of the transition pathways modelled."*

**R2.6**<br><br>Lines 528-520: Subsidy allocation "Windfall profits from mega-corporations reached 1.1 trillion USD in 2022 29in which we assumed are taxed by revenue68 Reviewer: 29 - Pages not found; 68 - The Internet address (url) you wanted to consult does not exist.	Excellent catch; indeed, the URL from Oxfam on the reference of the 1.1 trillion is no longer working, apologies for the inconvenience and thank you for pointing it out.<br><br>We have replaced it with a new one: *https://actionaid.org/news/2023/big-business-windfall-profits-rocket-obscene-1-trillion-year-amid-cost-living-crisis*<br><br>Please know that we have checked all references again for any inconsistencies.
**R2.7**<br><br>The equation 5 shows that calculation of the share of subsidy by country si doesn't represent the share of anything as a first part of this equation is equal to the following: (St($)*POPi*POPt)/(GDPi($)*GDPt($))<br>Where St is total subsidy; POPi is population in country i; POPt is total population; GDPi is GDP in country i ; GDPt is total GDP. Thus, si = 1/$ + % +Pi	We have re-written Equation 5 so that the calculations followed, which are available in Supplementary Data S2, are more clearly described (kindly see also comment R2.4).<br><br>Once again, let us thank the reviewer for their excellent feedback; we highly appreciate the insightful remarks and genuinely believe that the revisions made in response to both reviewers' reports have considerably enhanced the clarity and quality of our research.
